# High-speed atomic force microscopy reveals strongly polarized movement of clostridial collagenase along collagen fibrils

**DOI:** 10.1038/srep28975

**Published:** 2016-07-04

**Authors:** Takahiro Watanabe-Nakayama, Masahiro Itami, Noriyuki Kodera, Toshio Ando, Hiroki Konno

**Affiliations:** 1 Imaging Research Division, Bio-AFM Frontier Research Center, Kanazawa University, Kakuma-machi, Kanazawa 920-1192, Japan

## Abstract

Bacterial collagenases involved in donor infection are widely applied in many fields due to their high activity and specificity; however, little is known regarding the mechanisms by which bacterial collagenases degrade insoluble collagen in host tissues. Using high-speed atomic force microscopy, we simultaneously visualized the hierarchical structure of collagen fibrils and the movement of a representative bacterial collagenase, *Clostridium histolyticum* type I collagenase (ColG), to determine the relationship between collagen structure and collagenase movement. Notably, ColG moved ~14.5 nm toward the collagen N terminus in ~3.8 s in a manner dependent on a catalytic zinc ion. While ColG was engaged, collagen molecules were not only degraded but also occasionally rearranged to thicken neighboring collagen fibrils. Importantly, we found a similarity of relationship between the enzyme-substrate interface structure and enzyme migration in collagen-collagenase and DNA-nuclease systems, which share a helical substrate structure, suggesting a common strategy in enzyme evolution.

Collagenases secreted by bacteria promote infection and expansion by degrading collagen in the host tissues^1^,^2^. Collagen—the primary structural extracellular matrix component—is not a target for most proteolytic enzymes^1^. The unique structure of collagen consists of triple-helical monomers (tropocollagen molecules) that assemble into insoluble collagen fibrils, preventing normal proteinases from engaging^1^.

Bacterial collagenases are collagen-specific, zinc-dependent proteinases capable of degrading collagen in the host organisms^1^. The highly studied *Clostridium histolyticum* collagenases degrade collagen fibrils far more efficiently than vertebrate collagenases, known as matrix metalloproteinases (MMPs)^3^. Due to their specificity and high activity, clostridial collagenases are widely applied in various fields, including enzymatic wound debridement and fibroproliferative disorder therapy in healthcare, meat tenderization in the food industry, unhairing and dye diffusion processes in the leather industry, and the isolation of animal cells and tissues in scientific research^1^.

A variety of biochemical and structural studies have identified differences in the collagen hydrolysis mechanisms of bacterial and vertebrate collagenases. Clostridial collagenases cleave collagen at multiple sites, although they display preferences among the cleavable sites^4^,^5^, whereas MMP-1, MMP-8, and MMP-13 cleave at a single site 3/4 of the length from the N terminus of collagen^6^. Both clostridial and vertebrate collagenases bind multiple sites on collagen^7^,^8^ and cleave all three chains within a collagen monomer, albeit through different predicted mechanisms. The collagenase module of clostridial collagenase is large enough to surround a single tropocollagen molecule and to cleave all three chains at once^9^,^10^, a feature MMP-1 lacks. MMP-1 has no space to dock triple helical collagen chains and must instead unwind the helical chains near the cleavable site prior to cleavage^10^,^11^,^12^. Despite extensive research, the mechanism by which bacterial collagenases degrade insoluble collagen fibrils remains unclear. These analyses have been hindered due to the insolubility of collagen fibrils, although most collagen molecules are incorporated into the fibrils *in vivo*.

The disruption of soluble collagen monomers and the disruption of insoluble fibrils by bacterial collagenases are likely to occur through different mechanisms based on three considerations. First, although the collagen binding domains (CBDs) of clostridial collagenase are not necessary to process soluble collagen molecules^9^, they are required to bind insoluble collagen substrate^13^,^14^. Second, the assembly of monomeric collagen molecules into fibrils stabilizes the triple helical structure of individual monomers^15^,^16^. This stabilization is likely to affect CBD binding, as the clostridial class I collagenase (ColG) CBD binds the unwound region of a triple-helical collagen monomer^17^. Third, variations in the substrate collagen structure can alter the collagenolytic activity of other neighboring collagenases. MMP-1 activity is reduced by collagen fibril formation^3^,^18^. In collagen fibrils, the MMP-1-cleavable site is protected by an adjacent monomer’s C-terminal telopeptide, which must be removed by MMP-1 or other proteinases^19^. Fluorescence-labeled MMP-1 tracking indicates that these molecules move along collagen fibrils in a bidirectional, stepwise manner, where few of the stepwise movements are associated with collagenolytic activity^20^.

Here, we observed ColG engaged in collagen fibril degradation using high-speed atomic force microscopy (HS-AFM) to identify the mechanism by which ColG molecules migrate on and degrade type I collagen fibrils. Although fluorescence microscopy is usually adopted for single-molecule tracking experiments, there are several advantages for its use in HS-AFM. Importantly, HS-AFM allows the simultaneous video imaging of all objects on the microscope stage at nanometer resolution^21^. In this study, HS-AFM was used to visualize the movement of collagenase molecules and the structure of collagen fibrils concurrently. These analyses revealed the following features: (1) interactions between collagen molecules in the fibrils prevent ColG from engaging; (2) ColG molecules exhibit catalytic, zinc-dependent, polarized movement along the fibril axis toward the collagen N terminus, suggesting that ColG has efficiently coupled movement and function; and (3) while ColG degrades collagen, collagen molecules are occasionally rearranged and incorporated into neighboring fibrils. Thus, we obtained direct evidence of the dynamics of ColG and its substrate in the collagen fibril degradation mechanism. Furthermore, the collagenase-collagen interaction is similar to the nuclease-DNA system with respect to enzyme-substrate interface structure and enzyme movement, both of which involve the action of enzymes on the helical structure of substrates.

## Results

### Collagen fibril architecture

**Figure 1:**
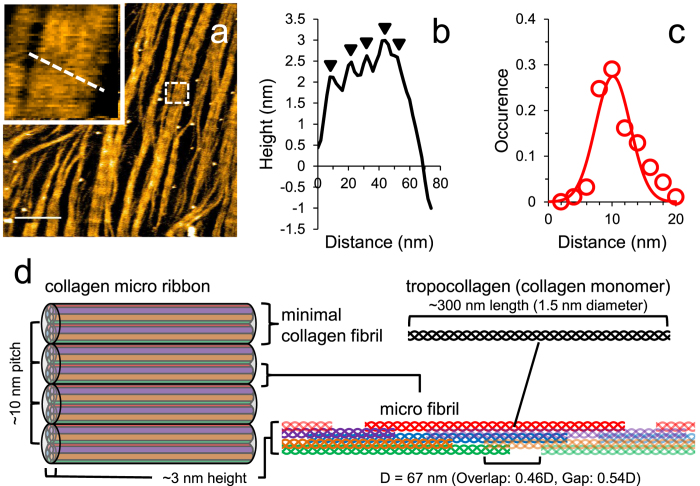
Collagen fibrils in a collagen microribbon on a HS-AFM stage. (**a**) HS-AFM image of collagen microribbon assembled from type I collagen of rat tail. These images are taken from Supplementary Movie 1. The region highlighted by a dashed box is enlarged in the inset, where five minimum collagen fibrils lie vertically. Scanning rate, 10 s/frame; Scan area, 1000 × 1000 nm^2^ with 500 × 500 pixels; Bar, 200 nm; Z-scale, 5 nm. (**b**) Height profile along the dashed line in the inset of (**a**). The peaks are indicated by arrowheads. (**c**) Distribution of the distance between collagen fibrils in collagen microribbon. (**d**) Schematic view of hierarchical structure of collagen microribbon. Five triple helical collagen molecule units (*red, blue, green, orange and purple*) associate into a microfibril with gaps and overlaps. Two microfibrils form a minimal collagen fibril. From this scheme, the number of collagen monomers is estimated to be 2975 per square μm.

Collagen molecules form self-assembled thin sheets characterized by D-bands, also known as collagen microribbons, on a mica surface in the presence of 0.2 M potassium^22^,^23^. Structurally, collagen microribbons resemble tubular collagen fibrils^22^. First, we confirmed that non-pepsin-treated rat tail collagen also formed these ~3 nm thick microribbons on mica in 50 mM Tris-HCl, 200 mM KCl, pH 7.5 (Fig. 1 and Supplementary Fig. 1a and Supplementary Movie 1). Rat tail type I collagen molecules were gradually aligned and assembled into a long, narrow sheet-like structure (Supplementary Fig. 1 and Supplementary Movie 1). The orientation of these assemblies can be controlled by hydrodynamic flow^23^, but the orientation was not controlled in our study. Consistently with previous research^22^, these sheets grew laterally in a stepwise manner with a length of ~4 nm, which is associated with microfibril formation (Supplementary Fig. 1). These microfibrils were then incorporated and rearranged^22^. We also observed the hierarchical structure of microribbon, in which incorporated collagen molecules were rearranged to form fibrils in the microribbon (Fig. 1a and Supplementary Movie 1). D-bands were observed in each fibril (Fig. 1a and Supplementary Movie 1), and the distance between adjacent fibrils was ~10 nm (Fig. 1b,c). These features in the individual fibril were consistent with a minimal collagen fibril assembled from two microfibrils that are each composed of five tropocollagen molecules^22^.

From these results, we estimated the number of collagen monomers per unit area. Five tropocollagen molecule units constitute a microfibril (Fig. 1d)^15^, and each collagen monomer (~300 nm in length) incorporates a gap and overlap of 0.54 D (36.18 nm) and 0.46 D (30.82 nm), respectively (Fig. 1d)^15^. The two microfibrils further assemble into a minimum collagen fibril ~10 nm in width and ~3 nm in height (Fig. 1d). The minimal collagen fibril of 336.18 nm in length (the sum of lengths of tropocollagen and the gap) and 10 nm in width is thus composed of ten collagen monomers. As ten collagen monomers cover an area of 3361.8 nm^2^, 1 μm^2^ of collagen microribbon contains ~2975 collagen monomers.

After the preparation of the collagen microribbons, the buffer solution was replaced with one suitable for collagenase assays. Although the electrolytes in the buffer affect the collagen alignment on mica^23^, collagen microribbons were observed in 50 mM Tris-HCl, 150 mM KCl, 10 mM CaCl_2_, pH 7.5 (TKC) with no observable differences (Supplementary Fig. 2).

### Video imaging of collagen fibril degradation by collagenases

**Figure 2:**
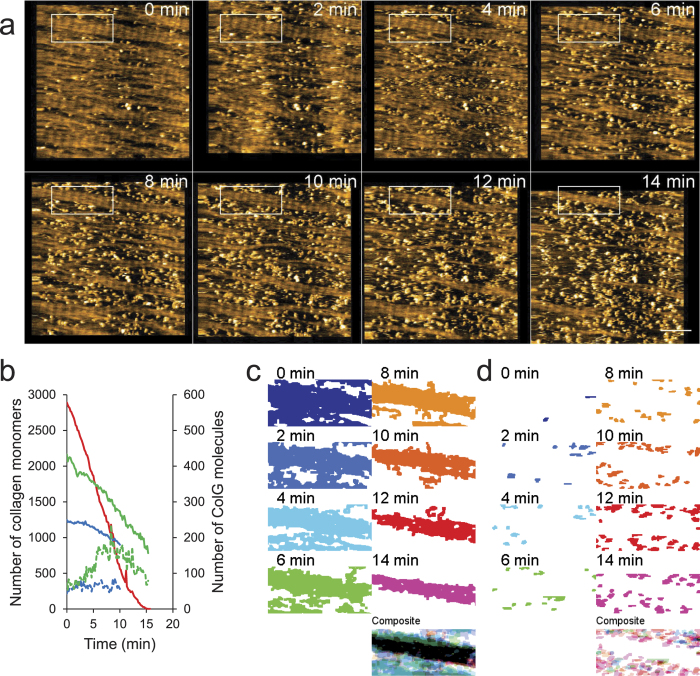
HS-AFM imaging of collagen fibril degradation by ColG. (**a**) Representative HS-AFM image sequence of collagen fibril degradation by 6 μg/mL ColG. These images are taken from Supplementary Movie 2. Scanning rate, 10 s/frame; Scan area, 1000 × 1000 nm^2^ with 500 × 500 pixels; Bar, 200 nm; Z-scale, 5 nm. (**b**) Number of remaining collagen molecules (*solid lines*) and number of collagenase molecules (*dashed lines*) at the edge of the collagen microribbon over time. Different colors correspond to individual experiments (1.5 μg/mL (*blue*), 6 μg/mL (*red* and *green*) ColG). (**c**,**d**) Binary image sequence and a composite image of the collagen-covered area (**c**) and ColG molecules (**d**) in the region highlighted in (**a**) show that microribbon is gradually degraded by ColG from the edge of the microribbon and that ColG molecules engage at the edge of the microribbon.

The addition of ColG molecules, which appeared as small particles, resulted in complete fibril degradation within a 20–30 minutes (Fig. 2 and Supplementary Movie 2). Decreases in collagen coverage correspond to the removal of collagen molecules from the microribbon. This process likely consists of multiple steps including cleavage event(s), fragment drop-off, and the unwinding of collagen molecules. Thus, there may be a time delay between the cutting and removal of fragments.

We estimated the turnover rate of ColG in collagen removal from microribbons. As shown in Fig. 2b, the amount of remaining collagen decreased proportionally to the time elapsed, whereas the number of ColG molecules on the collagen was nearly constant during each observation period, suggesting that ColG engagement on the collagen was associated with degradation of the collagen microribbon. The mean velocities and numbers of ColG molecules were then used to extrapolate the number of collagen monomers removed per ColG molecule per second. In the case of the *blue* and *green* curves in Fig. 2b, the average speeds of collagen degradation were 32.7 and 83.3 molecules/min/μm^2^, respectively, and the mean numbers of ColG molecules on collagen were 64 and 122 per μm^2^, respectively. Thus, we estimated the turnover rate of ColG for collagen fibril degradation (the number of collagen molecules removed by each ColG molecule per second), *k*_cat_ (/s), as ~0.0085/s for the *blue* curve and 0.0114/s for the *green* curve.

**Figure 3:**
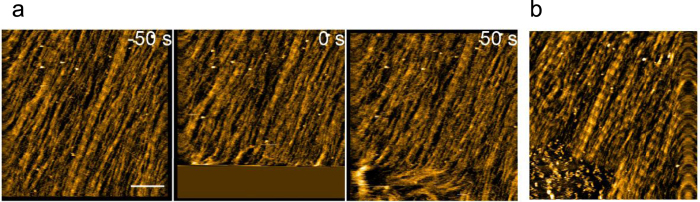
Disordered D-periodicity makes the collagen fibril susceptible to degradation by ColG. (**a**) HS-AFM image sequence of collagen microribbon before and after disturbing its alignment (see bottom left region in each frame) by a large tapping force. Scanning rate, 10 s/frame; Scan area, 1000 × 1000 nm^2^ with 500 × 500 pixels; Bar, 200 nm. (**b**) AFM image ~50 min after addition of 1.5 μg/mL ColG to the same area shown in (**a**). The disarrayed collagen is degraded faster than the ordered collagen fibrils (*bottom left*).

We then focused on the manner in which collagen microribbon was degraded by collagenases. Notably, ColG cleared the collagen microribbon from the lateral edge, rather than from the middle (Fig. 2c,d). To verify that the higher-order structure of collagen affected ColG activity, we intentionally disturbed the collagen alignment using a large tapping force applied by HS-AFM (Fig. 3a), followed by the addition of ColG. As shown in Fig. 3b, collagen clearance in the disturbed region was faster than in the adjacent D-periodic structure.

### Video imaging of individual ColG molecule movement on collagen fibrils

**Figure 4:**
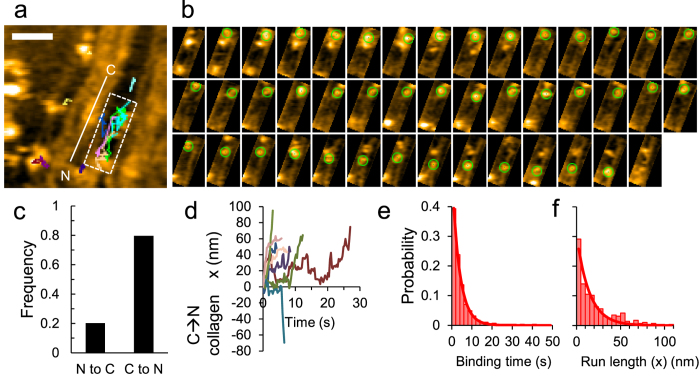
Strongly polarized movement of ColG on collagen fibrils. Representative traces (**a**) and successive AFM images (**b**) of the region highlighted in (**a**) show ColG movement from binding to dissociation. These images are taken from Supplementary Movie 3. Scanning rate, 0.3 s/frame; Scan area, 200 × 200 nm^2^ with 100 × 100 pixels; Z-scale; 5 nm; Bars, 50 nm. Time interval between images (**b**), 0.3 s. (**c**) Probability of the direction of net movement of ColG against the collagen fibril polarity. (**d**) Representative trajectories of ColG movement from binding to dissociation. (**e**,**f**) Distributions of the binding time (**e**) and the net run length of ColG movement parallel to the collagen fibril axis (**f**) for 285 molecules. Solid lines show single exponential fits, giving the apparent dissociation rate *k*_off_^app^ = 0.26 ± 0.00 s^−1^ and the mean run length *d*_x_ = 14.5 ± 1.5 nm.

To characterize the behavior of single collagenase molecules engaged in collagen microribbon degradation, we recorded image sequences at a higher spatiotemporal resolution. Fig. 4a, taken from Supplementary Movie 3, shows representative tracks of single ColG molecules on collagen fibrils from binding to dissociation. ColG molecules bound the lateral edge of collagen fibrils as described above and frequently slid along the collagen fibril axis. Several ColG molecules bound and migrated along the same collagen fibril, suggesting that one ColG engagement increased the binding affinity and/or catalytic efficiency for another at that location. Fig. 4b shows a representative image sequence of a single ColG molecule on a collagen fibril from binding to dissociation. The ColG molecule bound and paused, then moved unidirectionally along the fibril.

To define the polarity of ColG motion along the collagen fibril axis, we analyzed the polarity of individual collagen fibrils contiguous to ColG molecules. In the height profiles of collagen fibrils along the fibril axis, each overlap region in the D-bands shows an asymmetric peak^22^. We also confirmed the similar features and thus determined the polarity of the collagen fibrils (Supplementary Fig. 3). We were unable to discern the direction of short ColG movements. Moreover, ColG molecules tended to bind collagen fibrils in which the D-periodicity was unstructured; thus, we were unable to determine the sliding direction in many cases. Nevertheless, the migratory polarity of 69/285 molecules was clearly determined, with most ColG molecules (~80%) moving from the C to the N terminus (Fig. 4c).

To quantify ColG migration, we depicted the ColG position on axes parallel (x) and perpendicular (y) to the collagen fibrils, although ColG motion was always parallel. As shown in the time course (Fig. 4d), each ColG molecule moved unidirectionally along the collagen fibril axis at a non-constant speed. To characterize ColG migratory behavior on collagen, we analyzed the kinetics of ColG movement. As shown in Fig. 4e, the ColG residence time on collagen fibrils from binding to dissociation showed an exponential distribution, indicating that ColG migration and dissociation occurred randomly. The histogram can be fitted with a single exponential function, giving an apparent dissociation rate constant of *k*_off_^app^ = 0.26 ± 0.00 s^−1^ for ColG. Similarly, the distribution of the net run length for single engagement of ColG was also fitted with a single exponential function, giving a mean run length of *d*_x_ = 14.5 ± 1.5 nm (Fig. 4f). The mean run length (14.5 nm) and residence time (~3.79 s) (the reciprocal of apparent dissociation rate) of ColG are comparable to the turnover rate described above. As the collagen monomer length is ~300 nm, the rate of collagen removal (~0.01/s) can be converted to ~3 nm/s in length/time. Thus, the removed length during a single ColG molecule engagement (~3.79 s) was estimated to be ~11.3 nm. The similarity between the estimated values from the movement and functional analyses suggests high coupling efficiency between ColG movement and function.

To verify that these findings were due to ColG enzymatic activity, we also performed ColG collagenase assays in the presence of *o*-phenanthroline, which sequesters the catalytic zinc ion required for ColG function (apo-ColG). As shown in Supplementary Fig. 4a, collagen microribbon degradation did not occur in the presence of 1 mM *o*-phenanthroline, but removal of the chelator and subsequent addition of Zn^2+^-containing ColG (holo-ColG) restored the degradation activity. Thus, collagen microribbon collapse was the direct result of the hydrolytic activity of ColG. In addition, apo-ColG did not exhibit unidirectional sliding motion (Supplementary Fig. 4b–d and Supplementary Movie 4), and *k*_off_^app^ = 0.12 ± 0.01 s^−1^ for apo-ColG was approximately half the speed of holo-ColG (Supplementary Fig. 4e), suggesting that ColG catalysis is linked to dissociation.

### Video imaging of degradation of minimal collagen fibrils by ColG

**Figure 5:**
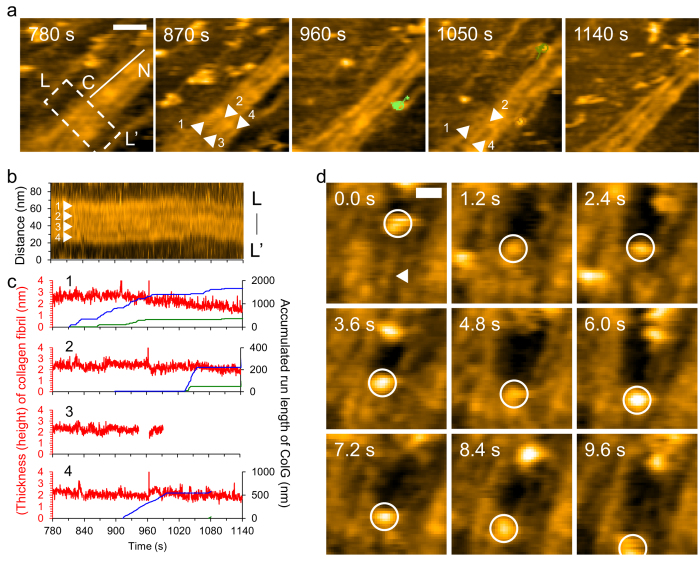
HS-AFM imaging of minimal collagen fibril degradation by ColG. (**a**) HS-AFM image sequence of minimal collagen fibril degradation by ColG. These images are taken from Supplementary Movie 5. The line with C and N denotes collagen fibril polarity. Colored circles with lines represent individual ColG molecules on collagen. The indicated times correspond to the elapsed times after the addition of ColG. Scanning rate, 0.3 s/frame; Scan area, 200 × 200 nm^2^ with 100 × 100 pixels; Bar, 50 nm; Z-scale, 5 nm. (**b**) Kymograph for the region highlighted in (**a**). Z-scale, 5 nm. (**c**) Time courses of thicknesses of individual minimal fibrils (*red*) indicated by numbers with arrowheads shown in (**a**,**b**), with accumulated ColG run length on individual fibrils within (*blue*) or including the outside (*green*) of the dashed-line box in (**a**). The total accumulated numbers of ColG molecules for given times on fibrils #1, #2, #3 and #4 are 11, 2, 0 and 1 within the region highlighted in (**a**) and 31, 3, 0 and 2 throughout the scan area of (**a**). (**d**) Successive HS-AFM images of disappearance of a minimal fibril (*arrowhead*) with the engagement of a ColG molecule (*circle*) (Supplementary Movie 6). Scanning rate, 0.3 s/frame; Scan area, 200 × 200 nm^2^ with 100 × 100 pixels; Bar, 20 nm; Z-scale, 5 nm.

To reveal how ColG removed a minimal collagen fibril, we focused on the relationship between the number of ColG engagements and the manner of degradation of individual minimal collagen fibrils. Fig. 5a shows four individual minimal fibrils under ColG engagements. Fibril #1 finally lost its D-bands at 1140 s, whereas fibril #3 disappeared at ~1000 s. From the kymograph of a single D-band of those fibrils (Fig. 5b), we analyzed the time courses of the thickness of each fibril and the accumulated ColG engagements on each fibril within that region and throughout the observed area (Fig. 5c). Fibril #1 decreased in thickness from ~900 s at the rate of 0.32 nm/min. By contrast, other fibrils did not exhibit a continuous decrease in their thickness, except that fibril #3 suddenly disappeared at ~1000 s (Fig. 5c). ColG engagements concentrated on fibril #1 in these groupings, and thus the number of ColG runs was related to the collagen removal (Fig. 5c). However, the temporal changes were not necessarily correlated. For instance, fibril #1 continuously decreased in thickness without ColG between ~960 s and ~1050 s. This result may be due to two factors: (1) involvement of ColG movements far from the observed area and (2) a time delay between ColG engagement and collagen removal. Additionally, rearranged fibrils were still subject to ColG engagements. With the disappearance of fibril #3, fibrils #2 and #4 temporarily increased in thickness from ~970 s to ~1070 s and from ~1040 s to ~1080 s, respectively. Nevertheless, while a single ColG movement occasionally removed a collagen fibril from the microribbon (Fig. 5d), the number of ColG movements per degradation of a single minimal collagen varied. One reason was the persistent rearrangement of collagen fibrils in the microribbon.

## Discussion

Simultaneous video imaging by HS-AFM of the hierarchical structure of collagen and the collagenase migration provided insights into the chemomechanical mechanism of enzymatic insoluble collagen fibril degradation. In this study, we observed the following: (1) the interactions of inter-fibril collagen molecules prevented collagenase molecules from engaging; (2) collagen molecules were rearranged even when subjected to collagenase engagement; (3) ColG preferred to engage at the edge of the collagen microribbon; and (4) ColG traveled toward the collagen N terminus at a non-constant velocity, depending on its catalytic activity. From the obtained kinetic parameters, we found high efficiency in the coupling of ColG migration with function. The estimated collagen removal rate in this study (~0.01/s = ~0.6/min at room temperature) is approximately 3.5-fold the dissolution rate of fibrillar collagen by ColG in a bulk phase assay. Mallya *et al*. reported that the specific activity of ColG toward the dissolution of fibrillary rat type I collagen was 1600 μg collagen/min/mg ColG at 37 °C 24. This value can be converted to 0.61/min, whereby the molecular weights of ColG and collagen are regarded as 115,000 and 300,000 g/mol, respectively. The rate at 25 °C may be estimated as ~28.5% of the rate at 37 °C by interpolation of the Arrhenius equation for clostridum collagenase activity toward fibrillary type I collagen degradation^3^. Thus, the turnover rate was estimated to be 0.17/min at 25 °C based on previous studies. The difference in the current study may result from the difference in the alignment of collagen fibrils: 2-dimensional microribbons in the current study and 3-dimensional tubules in the previous work.

**Figure 6:**
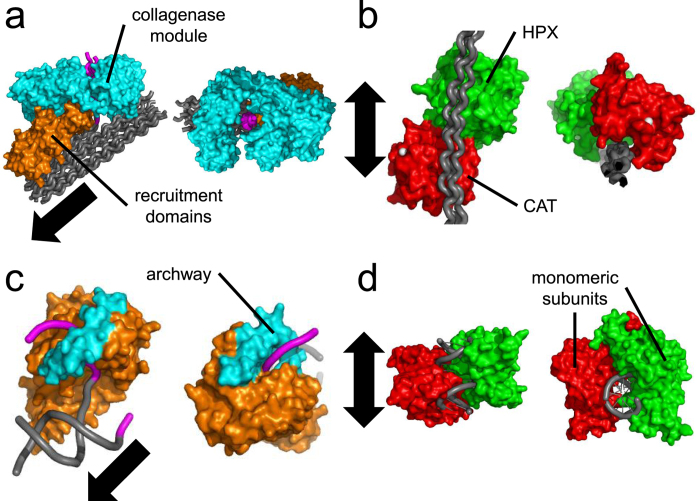
Comparison of collagenases and nucleases on their substrates with helical structure. (**a**) *Clostridium histolyticum* ColG on a collagen microfibril. The depicted structure is derived by positioning individual domains to satisfy biochemical studies (PDB ID code 4CLG, 2Y50, 4AQO, 1NQD). (**b**) Human MMP-1 on a triple helical collagen peptide (PDB ID code 4AUO). (**c**) T5 5′-exonuclease on a 5′ overhang double-stranded DNA (PDB ID code 1EXN aligned to 1J5F by PyMol). (**d**) Restriction endonuclease, *Eco*RV, on a double-stranded DNA (PDB ID code 2B0D). All of the enzyme-substrate complexes are represented as pairs of top (*left*) and side (*right*) views. Arrows indicate movement directions of the individual enzymes. *Gray* and *magenta* ribbons correspond to the wound and stripped regions of helical substrates. The domains colored *cyan* and *orange* correspond to the catalytic and substrate-binding domains. *Red* and *green* indicate the CAT and HPX domains of MMP-1 and the individual dimeric subunits of *Eco*RV.

The observed polarized movement of ColG is consistent with the chemomechanical model predicted based on structural and biochemical studies. ColG binds collagen mainly via its recruitment unit, which includes two CBDs, in cooperation with its N-terminal collagenase unit^14^ (Fig. 6a). The CBD is essential for collagenase binding to insoluble collagen^14^ and plays a role in the unidirectional docking of ColG on collagen^25^. CBDs are mainly composed of several β-sheets (PDB ID code 1NQD)^26^, which align perpendicularly to the collagen chains and provide the amino acid residues involved in collagen binding (CBD-collagen docking model in ref. 25). In the ColG turnover cycle, while the saddle-shaped N-terminal collagenase unit (Fig. 6a) undergoes a repeated open-close conformational change to unwind and cleave triple-helical collagen, the recruitment module maintains binding to prevent the enzyme from dissociating from the substrate^9^. In this way, ColG is predicted to increase processivity^9^. Additionally, the ColG collagenase unit has both endopeptidase and tri-carboxypeptidase activities^27^. The wall-like loop in the active site serves as a molecular ruler for substrate recognition to provide the tri-carboxypeptidase activity^9^. Thus, ColG has been predicted to move toward the collagen N-terminus^9^. There have been two candidate energy sources for driving ColG movement: (1) Ca^2+^-dependent conformational change in CBD and (2) entropic increase through the release of water molecules from the hydration layer on collagen in collagen cleavage^9^.

The short run length of ColG may be due to the hierarchical structure of collagen. This idea is supported by our observation that interactions between collagen molecules limit collagenase accessibility and activity (Figs 2c,d and 3). The packing of collagen molecules in fibrils stabilizes their helical structure, as described above^15^,^16^, which suggests that collagen fibril formation may hide collagenase-accessible sites and reduce the efficiency of collagen stripping by the ColG collagenase module. D-periodic collagen fibrils are difficult for ColG molecules to degrade, but once collagen monomers in fibrils are cleaved by ColG, the fibrils become unstructured, lose their D-bands, and thus become more susceptible to ColG engagement.

Enzyme processivity is also related to the substrate-enzyme interface structure. In the chitin-chitinase system, the enzyme run length (involved in processivity) is closely related to the ratio of the numbers of binding sites for the substrate and product^28^. The long substrate-binding site is a key factor in the high processivity of chitinases^28^. However, the kinetics of full-length ColG-collagen fibril binding remains unclear due to its complicated binding manner^14^.

In addition, the crossover of microfibrils may tighten the hierarchical collagen structure and inhibit the exposure of full-length collagen molecules at the edges of collagen fibrils. Tubular collagen fibrils are integrated by closely and intricately entangled microfibrils oriented at ~15° relative to the D-band^29^,^30^,^31^,^32^,^33^,^34^,^35^. Consistent with the tubular collagen fibrils, the ~15° tilt is confirmed in collagen microribbons on mica^22^. In the present study, crossover between the minimum collagen fibrils was observed (Supplementary Fig. 3). The alignment of individual collagen fibrils at a ~15° tilt suggests that the collagen microribbon has polarities in the longitudinal and latitudinal axes. ColG prefers to first cleave a C-terminal site, second process an N-terminal site, and then cut the internal sites of soluble type I collagen molecules^4^,^5^. ColG was expected to unidirectionally degrade collagen microribbon from one lateral side; however, it degraded collagen microribbon from both lateral edges. The stripping of collagen occurs at the regions near both termini in the higher-order collagen structure.

Meanwhile, MMPs, which share a common substrate with clostridial collagenases, have been known to display a diffusive motion on collagen^36^,^37^. The direction of diffusion along collagen is biased by the collagenolytic activity, which is explained by the “burnt bridge Brownian ratchet”^36^,^37^. Single-molecule observation reveals that MMPs bind to various regions on the collagen molecule^7^,^38^ and that MMP-1 exhibits bidirectional, stepwise motion parallel to the collagen fibril axis^20^ (Supplementary Information and Supplementary Figs 5 and 6). Most of the steps are independent of cleavage events^20^. Although the precise structural basis for MMP-1 motion on collagen is presently unknown, the difference in the substrate-enzyme interface between ColG and MMP-1 may produce these distinct movements. Manka *et al*. found that at the MMP-1 interface on the cleavable site of triple helical collagenous peptides, the active form of MMP-1 binds a collagen monomer cooperatively with its N-terminal catalytic (Cat) and C-terminal hemopexin-like (Hpx) domains, which are connected by a flexible linker region^39^ (Fig. 6b). The crystal structure shows that the two domains are arranged in a nearly straight line on the collagen molecule (Fig. 6b). In contrast to ColG, both the HPX and CAT domains of MMP-1 bind to collagen without surrounding it. The amino acid residues involved in collagen binding are mainly on loop-like structures, which align parallel to the collagen chains (PDB ID code 4AUO). The differences between ColG and MMP-1 are summarized in Table 1.

Comparing our findings with other enzymes suggests a common relationship between enzyme activity and enzyme-substrate interface structure. The relationship between collagen and collagenases is similar to that of DNA and nucleases. Collagen and DNA share a hydrated helical structure, and MMP-1 and ColG on collagen correspond to DNA-bound type II restriction endonucleases (REs) and flap endonucleases (FENs), respectively. Similar to the zinc-requirement of collagenases, these nucleases require divalent metal ion(s) to hydrolyze DNA^40^.

FENs recognize specific structures rather than particular DNA sequences and have a variety of nuclease activities, including flap single-stranded DNA (ssDNA) removal and 5′-3′ exonuclease activities against double-stranded DNA (dsDNA)^41^. These features resemble ColG’s multiple scissions and unidirectional movement. FENs consist of a dsDNA-binding domain and an arch-like catalytic domain that docks to ssDNA (threading model)^41^ (Fig. 6c), which correspond to the recruitment domains and the collagenase module of ColG, respectively (Fig. 6a,c).

Meanwhile, some type II REs (*Eco*RV, *Apa*I) bidirectionally diffuse and recognize particular cleavage sequence(s) in dsDNA^42^,^43^, similar to that observed for MMP-1. Additionally, the type II RE-dsDNA interface is similar to that of MMP-1-collagen. As shown in Fig. 6d, *Eco*RV rides on dsDNA rather than surrounding it, which is identical to MMP-1 on collagen.

HS-AFM allowed the direct and simultaneous analysis of the hierarchical structure of collagen and collagenase migration to reveal the relationship between the direction of collagenase movement and the substrate orientation, thereby clarifying the underlying chemomechanical coupling mechanism. However, there is a limitation in the traceable speed and size of enzyme motions because the image acquisition rate and scanning range are in a reciprocal relationship (as described in Materials & Methods)^44^. Thus, HS-AFM may not have the combination of speed and range required to resolve large-scale motions. Nevertheless, the high spatiotemporal resolution of HS-AFM makes it a powerful tool for exploring other chemomechanical systems involved in the higher-order structure of biomolecules. Higher-order molecular structures with a variety of hierarchical arrangements are ubiquitous in living organisms; for example, the chromosome, cytoskeleton, cellular membrane and cell wall. The activities of the enzymes involved may be associated with the hierarchical level of the substrate structures. However, these structures are usually insoluble, which have prevented us from understanding the relationships between the substrate structure and the enzyme movement. Nanometer-scale video imaging by HS-AFM sheds light on such relationships.

## Methods

### Buffers

Buffer A: 25 mM Tris-HCl, 50 mM NaCl, pH 7.5; Refolding buffer: 1 M arginine, 50 mM Tris-HCl, 150 mM NaCl, 5 mM CaCl_2_, 0.1 mM ZnSO_4_, pH 7.5 containing 2.5 mM each of reduced/oxidized glutathione; TK: 50 mM Tris-HCl, 200 mM KCl, pH 7.5; TNC: 50 mM Tris-HCl, 150 mM NaCl, 10 mM CaCl_2_, pH 7.5; TKC: 50 mM Tris-HCl, 150 mM KCl, 10 mM CaCl_2_, pH 7.5; TNKC: 50 mM Tris-HCl, 150 mM NaCl, 25 mM KCl, 10 mM CaCl_2_, pH 7.5; TNC1: 50 mM Tris-HCl, 150 mM NaCl, 1 mM CaCl_2_, pH 7.5.

### Preparation of collagenases

Collagenases were prepared using an *Escherichia coli* recombinant protein expression system. The *Clostridium histolyticum* genomic DNA fragment encoding collagenase G (ColG) without the propeptide was inserted into the pET15b vector (Novagen, Madison, WI) using the In-Fusion system (Takara, Otsu, Shiga, Japan). In the expression plasmid DNA, the ColG open reading frame (ORF) was fused to a Strep-tag II and a hexa-histidine tag (His-tag) at the N and C termini, respectively. Human MMP-1 cDNA, except for the region coding the signal peptide, was inserted into the pET15b vector and fused to a His-tag at the N terminus.

The recombinant collagenases were expressed in *Escherichia coli* BL21(DE3). Recombinant ColG protein was purified from the supernatant of the cell lysate using two successive affinity resins (HisGraviTrap [GE Healthcare Life Sciences, Tokyo, Japan] and Strep-Tactin Superflow high-capacity [IBA GmbH, Goettingen, Germany]), and then treated with enterokinase to release the N-terminal Strep-tag II. The recombinant ColG was further purified by gel filtration on a Superdex^TM^ 200 10/300 GL (GE Healthcare Life Sciences, Tokyo, Japan) in buffer A. Although the N-terminal His-tag alters the *N*-(3-[2-Furyl]-Acryloyl)-Leu-Gly-Pro-Ala (FALGPA) peptide digestion activity of ColG^45^, little difference was observed in collagenolytic activity, regardless of the N-terminal Strep-tag (Supplementary Fig. 7a and Supplementary Table 1). Recombinant pro-MMP-1 localized to the inclusion bodies and was solubilized in 8 M guanidine-hydrochloride supplemented with 100 mM DTT. The solubilized protein was then refolded by 100-fold dilution in refolding buffer and incubation for 1 day. Arginine, zinc, and reduced/oxidized glutathione in the sample solution were removed by dialysis against TNC buffer. The solubilized pro-MMP-1 was then concentrated and purified using ultrafiltration and Ni-NTA agarose resin (Qiagen, Hilden, Germany). Pro-MMP-1 was activated by TPCK-trypsin (Thermo Scientific), which cleaved the pro-MMP-1 propeptide. Active MMP-1 was purified by gel filtration on a Superdex^TM^ 75 (GE Healthcare Life Sciences, Tokyo, Japan) in buffer A. The purified active MMP-1 was concentrated by ultrafiltration. Both purified recombinant proteins were flash-frozen in liquid nitrogen and stored at −80  °C.

### Collagenase assay in bulk solution

The collagenolytic activity of ColG in bulk solution was examined by the digestion of fluorescein-conjugated collagen, DQ^TM^-collagen (type I collagen from bovine skin, Life Technologies, Carlsbad, CA), at 25 °C (Supplementary Fig. 7a,b). The time courses of the fluorescence intensity, *F*(*t*), were well fitted by the following double exponential function:


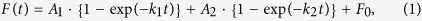


where *A*_1_ and *A*_2_ denote constants, and the sum of these(*A*_1_ + *A*_2_) is related to the concentration of the fluorescence-conjugated collagen, *k*_1_ and *k*_2_ are rate constants, and *F*_0_ corresponds to the background level of the fluorescence intensity. The increase in fluorescence produced by MMP-1 was much lower than for ColG. Instead, MMP-1 activity in bulk solution was analyzed by SDS-PAGE (Supplementary Fig. 7c–f, Supplementary Table 2). The solution condition is described in the caption of Supplementary Fig. 7.

### High-speed AFM imaging

A high-speed atomic force microscope was equipped with a small cantilever (BL-AC10-DS-A2 [Olympus, Tokyo, Japan]: spring constant, *k* = 0.1 N/m, resonance frequency, *f* = 400 ~ 500 kHz in water) and was operated in tapping mode at room temperature^21^,^44^. An amorphous carbon tip was grown on the top of each cantilever by electron-beam deposition with a scanning electron microscope (ERA-8000FE [Elionix, Tokyo, Japan]). The free oscillation amplitude was 1.4 ~ 2 nm, and the typical set-point amplitude was 85% of the free oscillation amplitude. A mica disk 1 mm in diameter fixed by epoxy glue on a glass rod 2 mm in diameter and 2 mm in height was used as a sample stage^44^. Before or after the sample stage was fixed by nail polish on the z-piezo of the HS-AFM scanner, the mica was freshly cleaved. This scanner was then mounted above the sample chamber with a cantilever immersed in TK buffer. After imaging the mica surface, the TK buffer was replaced by TK buffer containing 9 or 19 μg/mL rat tail type I collagen (Sigma, St. Louis, MO, USA)^22^,^46^. The chamber solution was typically replaced by removing the solution using a special pipette connected to a pump, followed immediately by the addition of suitable solution. Collagenolysis by the recombinant collagenases on mica was observed after replacement of the chamber solution with one of the following conditions: TKC with/without 1 mM *o*-phenanthroline for ColG or TNKC for MMP-1. The buffer solution for HS-AFM was developed as described in the Supplementary Information (Supplementary Figs 7,8 and 9). The microribbon structure was stably retained for at least ~1 hr in the assay buffer, as no significant structural disorder was observed within that time.

The image acquisition rate was determined as follows. The time, *T*, required for the acquisition of one frame (time resolution) in AFM imaging is represented by the following equation:


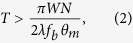


where *W*, *N*, *f*_b_, *λ* and *θ*_m_ represent the scan range in the x-direction, the number of scan lines, the feedback bandwidth, the smallest sample surface corrugation to be observed, and the sample fragility-dependent allowable maximum phase delay in the feedback control, respectively^44^. For the HS-AFM observation of biomolecules, *f*_b_ and *θ*_m_ are typically 110 kHz and 20°, respectively. Under the imaging conditions for this study (typically, *W* = 200 nm, *N* = 100, *λ* = ~5 nm), this equation estimates *T* > 164 ms for a one-frame acquisition. Consistently, we successfully observed ColG movement on a collagen microribbon at 250–300 ms/frame. For the same imaging condition on mica in TKC, most single ColG molecules stayed in the observed area for only a few frames and immediately moved out of the area (diffusion coefficient, *D* = ~1200 nm^2^/s) (Supplementary Fig. 8 and Supplementary Table 3).

### Image processing and data analysis

HS-AFM images were processed using the ImageJ software (National Institutes of Health, Bethesda, MD, USA). The drift of image sequences was corrected by the template matching plugin. To estimate the collagen coverage area, filters (variance, median, minimum) and/or a height threshold were used to eliminate collagenase molecules and exposed mica surface. For single-molecule analysis, the image sequences were processed using a 3 × 3 mean filter, and individual collagenases were tracked using the ImageJ plug-in MTrackJ^47^. The polarity of collagen was determined by assessing the D-bands in the collagen fibrils (Supplementary Fig. 3)^22^. For the topographical analysis, we used the average of consecutive video frames for each collagen fibril on which each collagenase moved. For quantitative analysis of collagenase movement, each motion vector was divided into two components parallel (x) and perpendicular (y) to each collagen fibril.

## Additional Information

**How to cite this article**: Watanabe-Nakayama, T. *et al*. High-speed atomic force microscopy reveals strongly polarized movement of clostridial collagenase along collagen fibrils. *Sci. Rep.* **6**, 28975; doi: 10.1038/srep28975 (2016).

## Supplementary Material

Supplementary Movie 1

Supplementary Movie 2

Supplementary Movie 3

Supplementary Movie 4

Supplementary Movie 5

Supplementary Movie 6

Supplementary Movie 7

Supplementary Information

## Figures and Tables

**Table 1:** Differences between bacterial and mammal collagenases on collagen degradation.

	Bacterial type collagenase	Mammal type collagenase
Collagen type specificity	Broad^1^	Type specific^48^
Cleavable site(s)	Multiple sites^4^,^5^ (N-terminal side of glycine residues)	Single site^6^,^49^ (3/4 length from N-terminus)
Cleavage manner	Cleave all 3 chains at once^9^,^10^	Cleave 3 chains one by one^10^,^11^,^12^
Movement along collagen fibrils	Strongly polarized* (C- to N-terminal side of collagen)	Biased bidirectional stepwise^20^,^37^

^*^The present study.
