# Supplementary material for: High-speed atomic force microscopy reveals strongly polarized movement of clostridial collagenase along collagen fibrils
Source: Sci Rep. 2016 Jul 4;6:28975. doi: 10.1038/srep28975 (PMC4931465; doi:10.1038/srep28975)
Supplement: Supplementary Information [file srep28975-s8.pdf]

Supplementary Information for
High-speed atomic force microscopy reveals strongly polarized movement of clostridial collagenase along collagen fibrils

Takahiro Watanabe-Nakayama, Masahiro Itami, Noriyuki Kodera, Toshio Ando, and Hiroki Konno

Imaging Research Division, Bio-AFM Frontier Research Center, Kanazawa University

Supplemental Inventory

1. Collagen microribbon.
   - Supplementary Fig. 1. Growth of collagen microribbon on mica.
   - Supplementary Fig. 2. Structural details of collagen microribbons on mica.
   - Supplementary Fig. 3. Polarity of collagen fibrils in a microribbon.
2. HS-AFM observation of ColG on collagen microribbon in the presence of an inhibitor.
   - Supplementary Fig. 4. Zinc-depleted ColG on collagen fibrils.
3. HS-AFM observation of MMP-1 on a collagen microribbon.
   - Supplementary Fig. 5. HS-AFM imaging of collagen fibril degradation by MMP-1.
   - Supplementary Fig. 6. Bidirectional stepwise MMP-1 motion parallel to the collagen fibril axis.
4. Adjustment of buffer solution for HS-AFM imaging.
   - Supplementary Fig. 7. Collagenase activity in bulk solution.
   - Supplementary Fig. 8. Monovalent cation-dependent collagenase mobility on mica.
   - Supplementary Fig. 9. Monovalent cation-dependent collagenase activity and mobility on mica.
   - Supplementary Table 1. Kinetic parameters for ColG from fluorescence-labeled collagen degradation assay.
   - Supplementary Table 2. Kinetic parameters for prepared MMP-1 activity toward soluble type I collagen.
   - Supplementary Table 3. Diffusion coefficients (nm^2/s) for ColG, MMP-1 and particles in collagen samples in different buffers on mica or collagen.
5. Captions for Supplementary Movies.
   - Supplementary Movie 1.
   - Supplementary Movie 2.
   - Supplementary Movie 3.
   - Supplementary Movie 4.
   - Supplementary Movie 5.
   - Supplementary Movie 6.
   - Supplementary Movie 7.
6. Supplemental References.

1. Collagen microribbon.

As described in the Results section in the main text, rat tail type I collagen assembled into microribbons on mica in TK buffer with a manner of growth similar to a previous study [1] (**Supplementary Fig. 1**). After extensive growth of the microribbon, the solution in the sample chamber was replaced by a collagenase assay buffer. Because the structure of collagen microribbon is affected by electrolytes in the buffer solution [2], we analyzed whether the replacement of buffer solution altered the microribbon structure. As described in the main text, no apparent difference was observed between TK and TKC (**Supplementary Fig. 2**). However, in TNKC (mainly used for the MMP-1 assay described below), the collagen microribbon was rearranged, although D-bands were still observed (**Supplementary Fig. 2**). The height of collagen microribbon was reduced to ~1.5 nm, which corresponds to the height of a tropocollagen molecule (**Supplementary Fig. 2c**) [3], and the collagen coverage area was doubled (**Supplementary Fig. 2f-g**). The distance between adjacent fibers was slightly reduced to ~ 9 nm (**Supplementary Fig. 2c-d**). Based on the structural features of the altered microribbon, the number of collagen monomers per unit area in the TNKC buffer was reduced by half. Thus, we estimated the number of collagen monomers in TNKC buffer to be ~1487/μm^2 collagen microribbon.

As shown in **Supplementary Fig. 2a-b** (*arrowheads*), some globular particles were observed during the preparation of collagen microribbon even in the absence of collagenase. There are two possibilities for the identity of these particles: contamination or the misfolding products of collagen molecules. Collagen molecules change their structure in response to pH [2]. Under acidic conditions (stock solution), collagen molecules exhibit globular structure, whereas they extend to a string-like conformation in neutral and basic conditions [2]. These particles can be distinguished from collagenase molecules due to their extremely small diffusion coefficients (**Supplementary Figs. 2h** and **8h-i**, and **Supplementary Table 3**). Moreover, in HS-AFM imaging, they were present in much lower amounts than collagenase molecules.

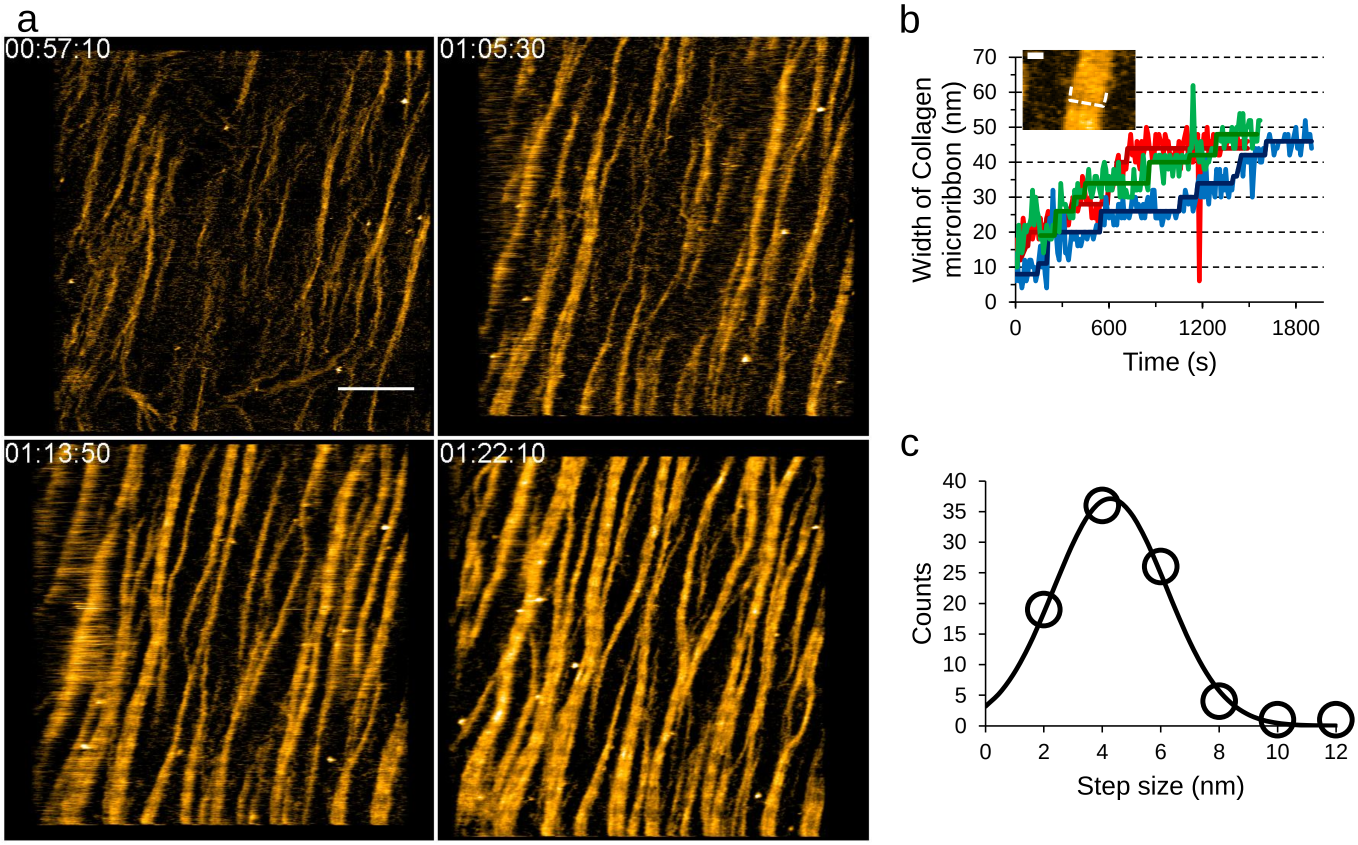

Supplementary Fig. 1. Growth of collagen microribbon on mica. (a) HS-AFM images of growing collagen microribbon from 57′10″ to 1º22′10″ after the addition of soluble type I collagen in TK to the sample chamber. These images are taken from **Supplementary Movie 1**. Scanning rate, 10 s/frame; Scan area, 1000 × 1000 nm^2, 500 × 500 pixels. (b) Representative time course of the width of the growing collagen microribbon. Different colors correspond to the different collagen microribbons. Darker lines show the median width of the ribbons in each dwell. The inset shows a representative growing collagen microribbon with a dashed line indicating its width. (c) Distribution of the step size in the growth in width of collagen microribbon with a Gaussian fit showing the mean step size, 4.3 ± 1.9 nm. Bars, 200 nm (a) and 20 nm (b). Z-scale, 5 nm.

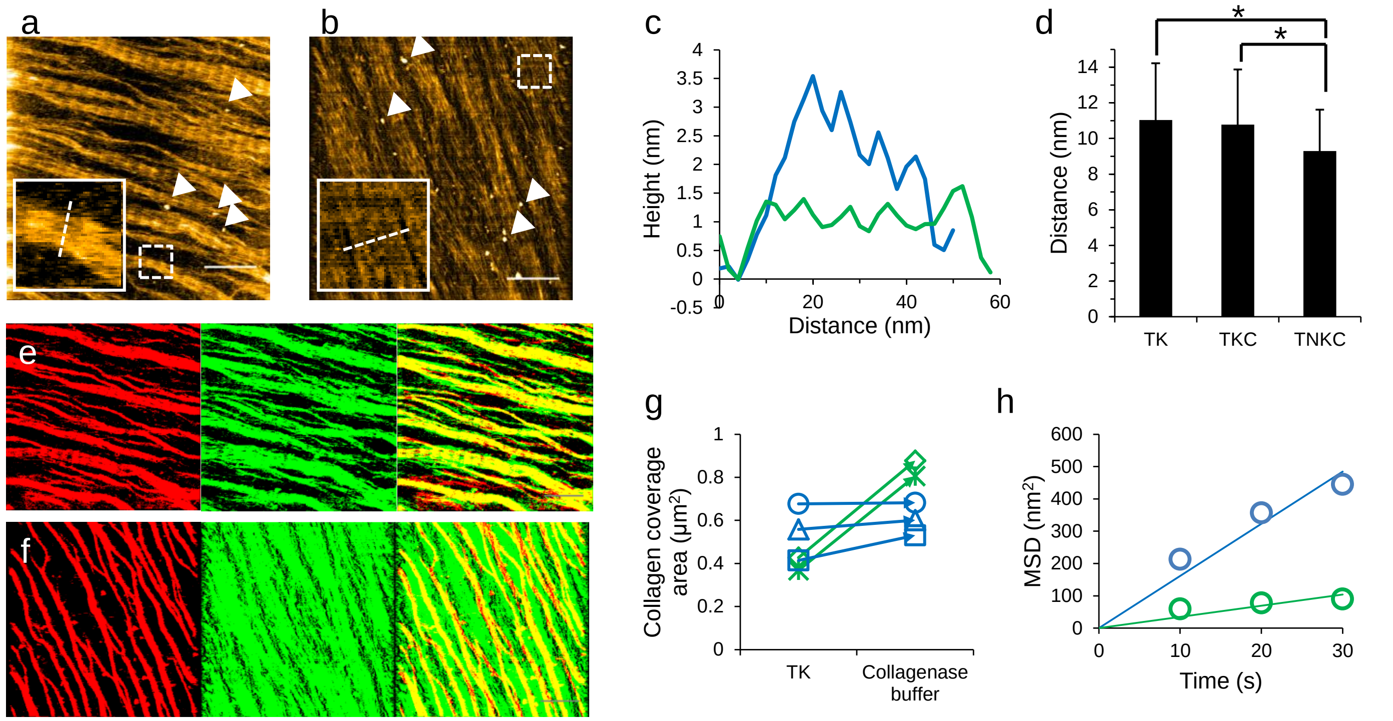

Supplementary Fig. 2. Structural details of collagen microribbons on mica. (a-b) HS-AFM images of collagen microribbons in TKC (a) or TNKC (b) buffer. The regions highlighted by dashed boxes are enlarged in the insets. (c) Height profiles along the dashed lines in (a) and (b), respectively shown as *blue* and *green lines*. (d) Distance between the peaks shown in the height profiles in (c). Bar graph shows mean values, and error bars indicate standard deviation: 11.0 ± 3.2 nm for TK; 10.8 ± 3.1 nm for TKC; 9.3 ± 2.3 nm for TNKC. Asterisks indicate $p < 0.005$ in t-tests. (e-f) Collagen coverage region before (*red* on *left*) and after (*green* in *center*) the replacement of TK with TKC (e) or TNKC (f). The right images are the merged left and center images. The *yellow* regions on the *right* correspond to the overlap regions before and after the replacement of buffer. (g) Collagen coverage area before and after the replacement of TK with TKC (*blue*) or TNKC (*green*). Different symbols correspond to different experiments. (h) Mean square distance plot with line fits (without offset) of diffusive movements of particles in collagen samples in TKC (*blue*) and TNKC (*green*). The representative particles are indicated by *arrowheads* in (a) and (b). Scanning rate, 10 s/frame; Scan area, 1000 × 1000 nm^2 with 500 × 500 pixels; Bars, 200 nm; Z-scale, 5 nm.

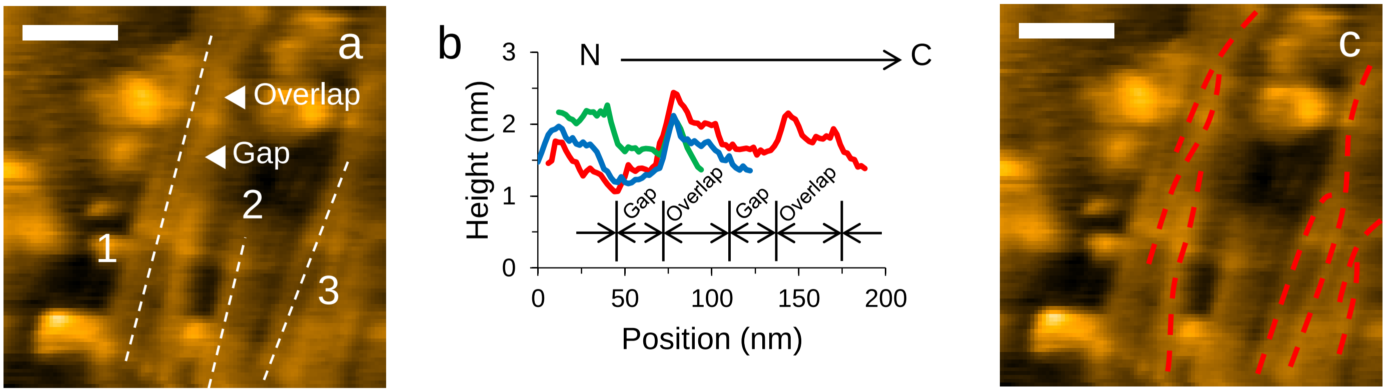

Supplementary Fig. 3. Polarity of collagen fibrils in a microribbon. (a) Average image of 30 successive frames of D-periodic collagen on mica. Scanning rate, 0.3 s/frame; Scan area, 200 × 200 nm^2 with 100 × 100 pixels. (b) Height profiles of the selected lines *1*, *2* and *3* in (a) are shown as *red*, *green* and *blue* curves, respectively. The profiles show an asymmetric peak at each of the overlap regions where the N-terminal side is higher than the C-terminal. (c) Alignment of individual minimal collagen fibrils (*red dashed lines*) in (a). Bars, 50 nm. Z-scale, 5 nm.

2. HS-AFM observation of ColG on collagen microribbon in the presence of an inhibitor.

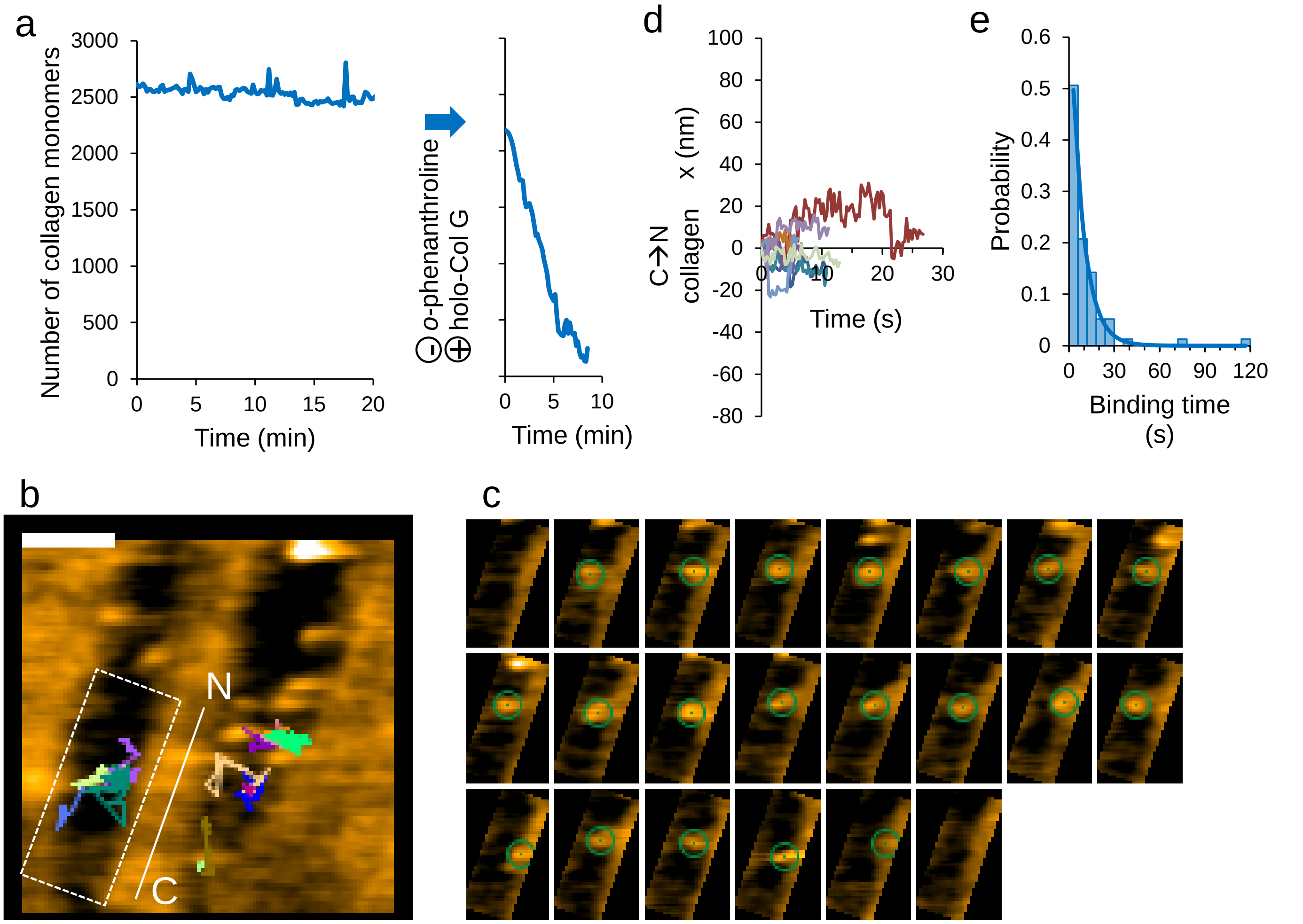

Supplementary Fig. 4. Zinc-depleted ColG on collagen fibrils. (a) Number of remaining collagen molecules over time in the presence of 1.5 µg/mL ColG with 1 mM *o*-phenanthroline (*left*): the degradation was restored by removal of the chelator (*o*-phenanthroline) and addition of holo-ColG (*right*). (b-c) Representative traces (b) and successive AFM images (c) of the regions highlighted in (b) show ColG movement from binding to dissociation in the presence of 1 mM *o*-phenanthroline. These images are taken from **Supplementary Movie 4**. Scanning rate, 0.3 s/frame; Scan area, 200 × 200 nm^2 with 100 × 100 pixels; Z-scale, 5 nm; Bar, 50 nm. Time interval between images (c), 0.3 s. (d) Representative trajectories of ColG movement from binding to dissociation in the presence of 1 mM *o*-phenanthroline. (e) Distribution of binding time in the presence of 1 mM *o*-phenanthroline (77 molecules). Solid line shows single exponential fit giving the apparent dissociation rate of 0.12 ± 0.01 s^{-1}.

3. HS-AFM observation of MMP-1 on a collagen microribbon.

We performed a comparative analysis of collagen microribbon degradation by MMP-1 and ColG. **Supplementary Fig. 5** shows that MMP-1 molecules increased while the microribbon coverage area decreased (-3.37 and -0.97 collagen molecules/min/μm^2 for *red* and *blue* lines in **Supplementary Fig. 5b**). Similar to ColG, MMP-1 bound and degraded the microribbon from the lateral edge (**Supplementary Fig. 5c-d**); however, an excess of MMP-1 (15 μg/mL) was required, hindering our ability to quantify the bound enzyme. Nevertheless, the approximate turnover rate for prepared MMP-1 toward collagen microribbon was extrapolated from the number of bound MMP-1 molecules at 1.5 μg/mL (~204 molecules/μm^2) to be ~2.8×10^{-5}/s and ~7.9×10^{-5}/s (for the *red* and *blue lines* in **Supplementary Fig. 5b**). These values were ~100-fold lower than the turnover rate from ensemble measurements of fibril degradation (~0.005/s at 25ºC) [4–7]. This difference may be due to two factors: (1) the MMP-1 used in this study was activated by trypsin in the absence of plasmin and MMP-3 (The inactivated form, proMMP-1, which have propeptide at its N-terminus is fully activated by process of the propeptide by plasmin and MMP-3), supported by the difference in collagenase activity between prepared MMP-1 and the value reported in previous studies [4–7] (**Supplementary Fig. 7d-f, Supplementary Table 2**); and (2) the modified microribbon structure in TNKC reduced the collagenase activity (**Supplementary Fig. 2, Supplementary Fig. 9d-e**).

In the same way as for ColG, we analyzed the migration of individual MMP-1 molecules on collagen fibrils. **Supplementary Fig. 6a**, taken from **Supplementary Movie 7**, shows representative tracks of single MMP-1 molecules on collagen fibrils while they remained in the observed area. Similar to ColG, MMP-1 moved at the lateral edge of the collagen microribbon along the fibril axis; however, it showed different behavior from ColG. **Supplementary Fig. 6b** shows a representative image sequence of a single MMP-1 molecule on a collagen fibril from its appearance to its disappearance. The MMP-1 molecule moved bidirectionally along the fibril axis. As in the ColG analysis, motion vectors for MMP-1 are depicted on axes parallel (x) and perpendicular (y) to the collagen fibrils. The time course clearly shows that MMP-1 exhibited a bidirectional, stepwise motion along the fibril axis (**Supplementary Fig. 6c**). The step size and the dwell time before each step showed a single exponential distribution, giving mean step sizes of $d_f = 11.3 \pm 1.0$ nm for forward motion and $d_b = 12.0 \pm 0.9$ nm for backward motion (**Supplementary Fig. 6d**), and the mean dwell times before the step were $\tau_f = 0.54 \pm 0.02$ s for forward and $\tau_b = 0.39 \pm 0.02$ s for backward steps (**Supplementary Fig. 6e**). Statistical analysis revealed that MMP-1 migration occurred randomly. In addition, no significant differences were found between the forward and backward steps. The mean step size (~12 nm) was shorter than the distance between adjacent MMP-1 cleavable sites on a collagen microfibril (67 nm), and most individual steps did not result in collagen cleavage.

The results from our migratory analysis of MMP-1 are comparable in some respects to the results of a previous single fluorescence-labeled MMP-1 tracking experiment. This study found that the stepwise movement of MMP-1 includes at least two types of pauses—class I and II—which can be distinguished statistically [4]. Moreover, the dwell times for class I and II pauses show exponential and Gaussian distributions, respectively [4]. The majority of MMP-1 pauses correspond to class I, even at 37°C. At room temperature, the occurrence of the class II pauses—which involve MMP-1 catalytic activity—further decreases [4], although MMP-1 binding is not affected [9]. Our study performed HS-AFM at room temperature. The activity of prepared MMP-1 was intrinsically low due to incomplete activation, as described above. In addition, the altered microribbon structure present in TNKC may also reduce MMP-1 activity. These two factors could jointly reduce the activity compared to the enzyme used in the previous studies [4,10]. Thus, most of the observed steps for MMP-1 were representative of class I pauses, and biased diffusive motion was not observed

in this study.

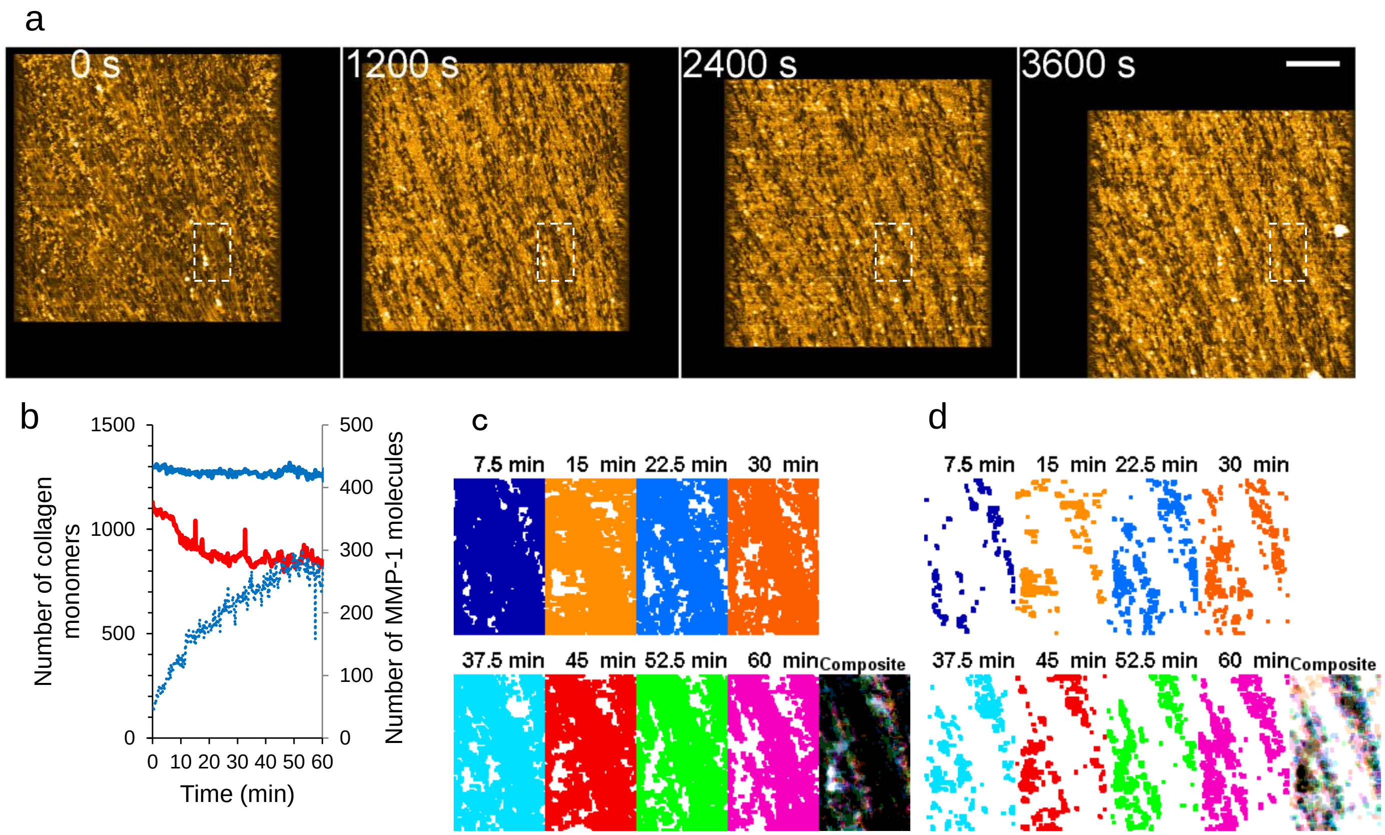

Supplementary Fig. 5. HS-AFM imaging of collagen fibril degradation by MMP-1. (a) Representative HS-AFM image sequence of collagen fibril degradation by 15 µg/mL MMP-1. Scanning rate, 10 s/frame; Scan area, 1000 × 1000 nm^2 with 500 × 500 pixels; Bar, 200 nm; Z-scale, 5 nm. (b) Number of remaining collagen molecules (*solid lines*) and number of MMP-1 molecules bound (*dashed line*) over time: *blue* and *red* correspond to 1.5 µg/mL and 15 µg/mL MMP-1. (c-d) Binary image sequences and composite images of the collagen-covered area (c) and MMP-1 molecules (d) highlighted in (a) show that the microribbon is slightly degraded by MMP-1 from the edge and that MMP-1 molecules engage at the edge of the microribbon.

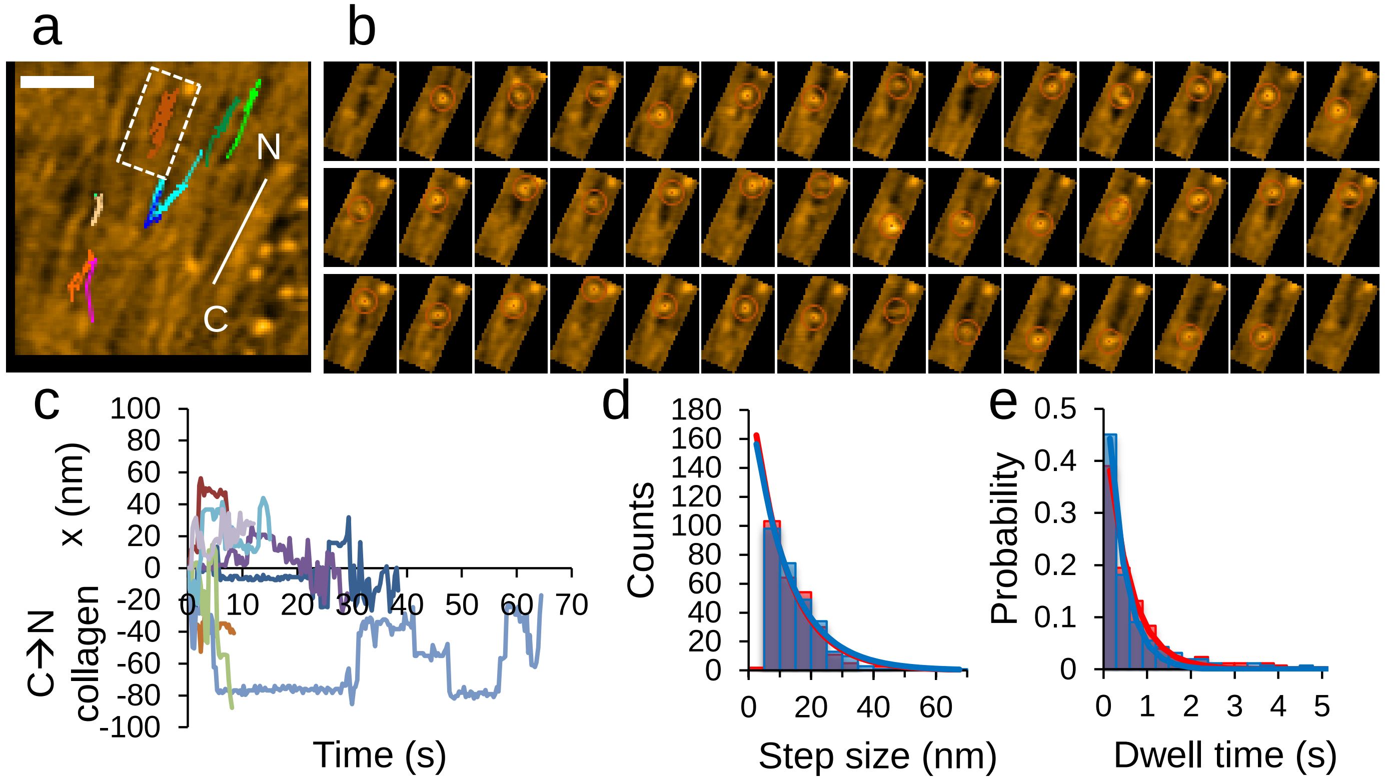

Supplementary Fig. 6. Bidirectional stepwise MMP-1 motion parallel to the collagen fibril axis. Representative traces (a), successive AFM images (b) highlighted by a dashed box in (a), and trajectories (c) of MMP-1 movement from appearance to disappearance. These images are taken from **Supplementary Movie 7**. Scanning rate, 0.3 s/frame; Scan area, 200 × 200 nm^2 with 100 × 100 pixels; Z-scale, 5 nm; Bar, 50 nm. Time interval between successive images, 0.3 s. (d) Distribution of step size in the forward (251 steps, *red*, C to N terminus of collagen) and backward (253 steps, *blue*) directions with single exponential fits (*solid lines*) giving a mean step size of $d_f = 11.3$ nm for the forward step and $d_b = 12.0$ nm for the backward step. (e) Distribution of the dwell time immediately before forward (*red*) and backward (*blue*) steps with single exponential fits (*solid lines*) giving a mean dwell time of $\tau_f = 0.54 \pm 0.02$ s before the forward step and $\tau_b = 0.39 \pm 0.02$ s before the backward step. Mann-Whitney U-tests comparing forward and backward steps showed that the differences in step size and dwell time were not significant at $p > 0.05$. The number of MMP-1 analyzed molecules was 68.

4. Adjustment of buffer solution for HS-AFM imaging.

We searched for an optimal condition in which collagenase molecules diffused extensively without strong interaction with mica or changes in enzymatic activity. Both ColG and MMP-1 strongly bound mica in TNC buffer, which is the general solution used for bulk collagenase assays (**Supplementary Fig. 8a,d,h-i**). ColG did not degrade the collagen microribbon on mica in TNC buffer (**Supplementary Fig. 9a,c**); however, we found that ColG exhibited diffusive movement on mica without alteration of its collagenolytic activity in TKC buffer. (**Supplementary Figs. 7b, 8b,h**, **9b-c and Supplementary Table 3**). TKC buffer also did not alter MMP-1 collagenolytic activity (**Supplementary Fig. 7c**), but MMP-1 molecules were not observed on mica in TKC buffer (**Supplementary Fig. 8e**). A small number of particles were observed on collagen microribbon in TKC containing MMP-1, but they failed to move (**Supplementary Fig. 9f,g**), suggesting that those particles are from the collagen sample (**Supplementary Fig. 2a,h** and **Supplementary Table 3**) and that most MMP-1 molecules did not approach the mica surface in TKC buffer, even in the presence of a collagen microribbon. Instead, we added a small amount of potassium to TNC buffer and checked both the MMP-1 diffusion on mica and its collagenolytic activity in bulk assays. The addition of a small amount of potassium ion (20–30 mM KCl) promoted MMP-1 diffusion on mica without significantly altering its activity (**Supplementary Figs. 7c-f, 8d,f-g,i**). The buffer solution selected for HS-AFM was TKC for ColG and TNKC for MMP-1.

By contrast, the substrate collagen microribbon structure was significantly altered in TNKC buffer, as described above (**Supplementary Fig. 2**). To assess the impact of this change on collagenase activity, we observed the microribbon degradation by ColG in TNKC buffer. The diffusion coefficient of ColG on mica in TNKC was lower than on mica in TKC but was still higher than on microribbon in TKC (**Supplementary Fig. 8** and **Supplementary Table 3**). As shown in **Supplementary Fig. 9d-e**, ColG degraded microribbon in TNKC, but its turnover rate was estimated to be less than in TKC. The number of ColG molecules on the stage surface in TNKC was apparently much larger than in TKC despite the addition of the same amount of ColG. Thus, the number of ColG molecules on microribbon was uncountable. Nevertheless, the degradation rate of microribbon in TNKC was lower (7.38 and 24.8 collagen molecules/min/μm^2 for *red* and *blue lines* in **Supplementary Fig. 9e**) than in TKC (32.7 molecules/min/μm^2 for *blue line* in **Fig. 2b** in the main text). Although the magnitude of this effect is not necessarily comparable for MMP-1, we confirmed that structural alteration in the substrate can affect MMP-1 activity on microribbons in TNKC.

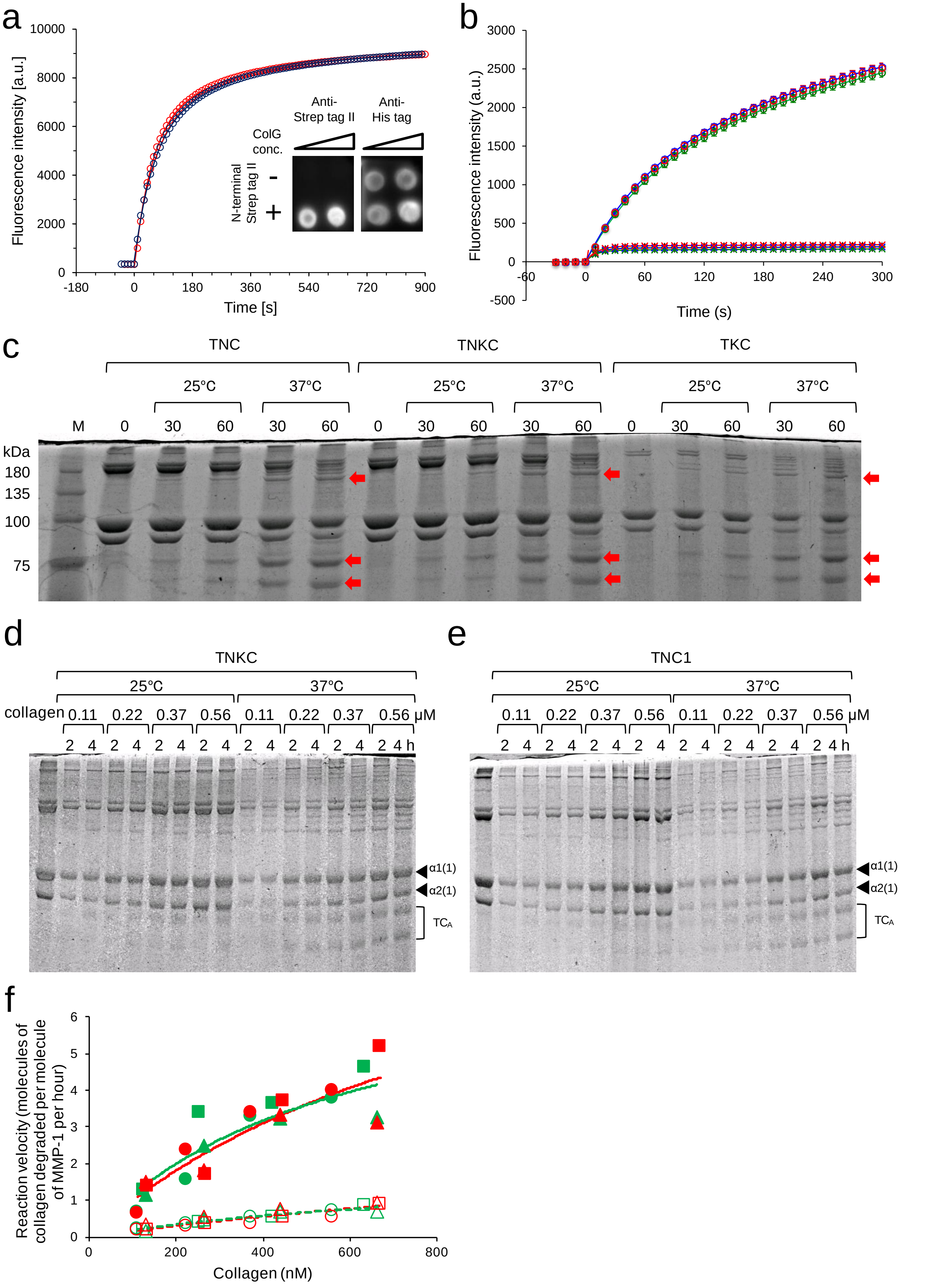

Supplementary Fig. 7. Collagenase activity in bulk solution. (a and b) Time course of fluorescence intensity for

fluorescein-conjugated collagen cleaved by ColG (*open circles* and *crosses*) with biexponential fits (*solid lines*) giving fitting parameters (**Supplementary Table 1**). Reactions contained 3 µg/mL (a) or 1.5 µg/mL (b) recombinant ColG and 0.05 mg/mL (a) or 0.02 mg/mL (b) fluorescein-conjugated type I collagen. (a) The reactions in TNC buffer were initiated by the addition of recombinant ColG with (*blue*) or without (*red*) the N-terminal Strep-tag II. The inset shows dot blot analysis of anti-Strep-tag II and anti His-tag antibodies against ColG with/without N-terminal Strep-tag II. (b) The reactions were conducted in TNC (*red*), TKC (*blue*) or TNKC (*green*) buffer in the absence (*circles*) or presence (*crosses*) of 1 mM *o*-phenanthroline. (c) Digestion of rat tail collagen by MMP-1. Rat tail type I collagen (0.09 mg/mL) was incubated with 9 µg/mL MMP-1 under the indicated conditions. The reactions were then subjected to SDS-PAGE in a reducing condition. Red arrows indicate the bands from collagen cleaved by MMP-1, which correspond to β_A, $TC^A_{\alpha 1}$ and $TC^A_{\alpha 2}$ from the top downward. The weak bands under TKC were due to precipitate caused by the addition of SDS-PAGE sample buffer. (d-f) Determination of K_m and k_{cat} for MMP-1 in degradation of rat tail type I collagen in TNKC and TNC1. (d-e) Formation of degradation product (TC_A) in TNKC (d) or TNC1 (e) containing 2 µg/mL MMP-1 at the indicated time, substrate concentration and temperature. The reaction was stopped by the addition of SDS-PAGE sample buffer. The amount of collagen degraded (%) was estimated from $4/3[TC_A]/(4/3[TC_A] + [\alpha])$. (f) Reaction velocity versus substrate concentration with Michaelis-Menten fits for the reaction in TNKC (*green*) or TNC1 (*red*) at 25°C (*open symbols* and *dashed lines*) or 37°C (*closed symbols* and *solid lines*). Different symbols correspond to individual experiments. The K_m and k_{cat} values determined are shown in **Supplementary Table 2**.

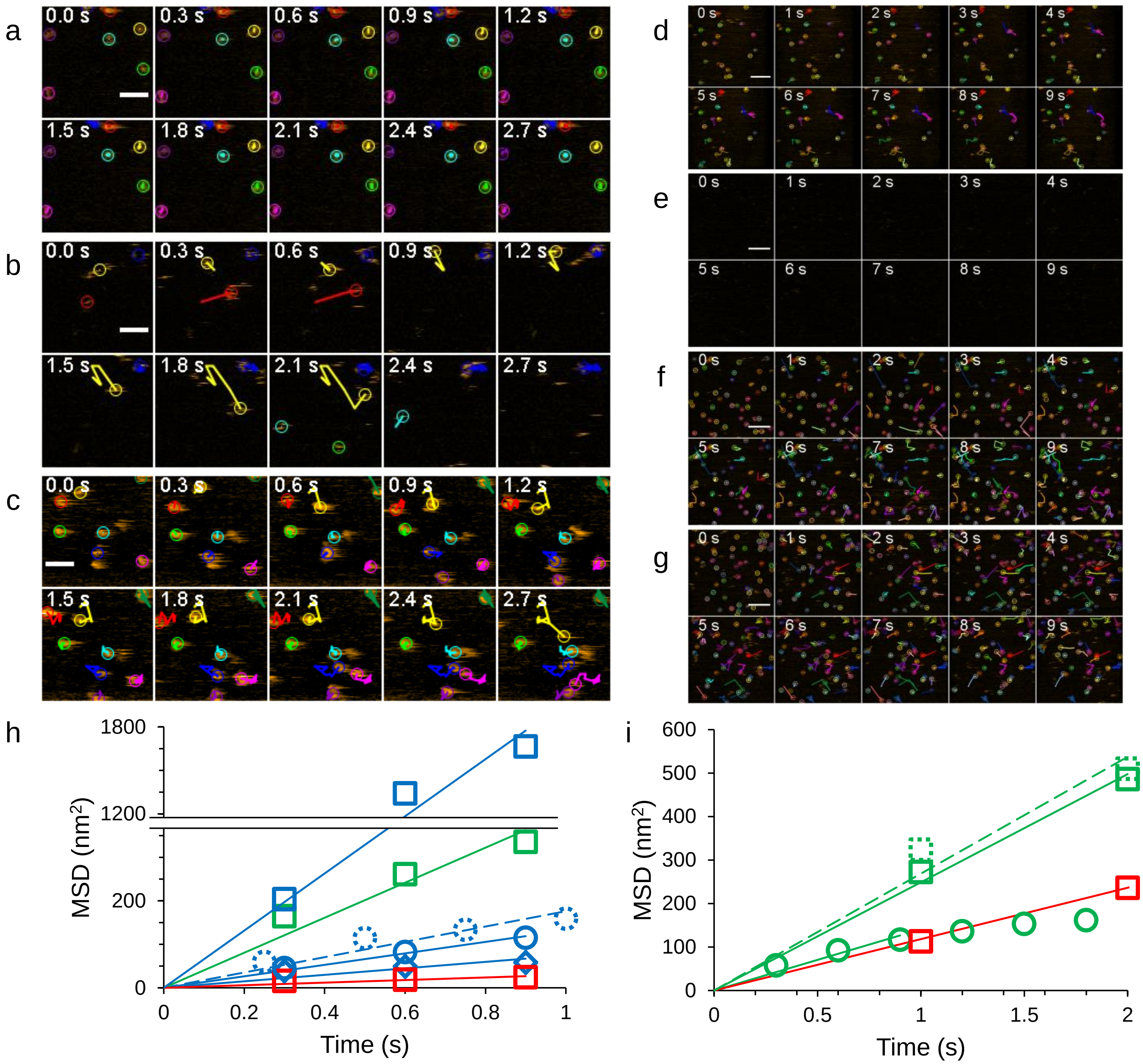

Supplementary Fig. 8. Monovalent cation-dependent collagenase mobility on mica. (a-g) Successive 10 HS-AFM images showing mobility of ColG (a-c) and MMP-1 (d-g) on mica in TNC buffer (a and d), TKC buffer (b and e) and TNC buffer containing KCl at 20 mM (f), 25 mM (c) or 30 mM (g). The different colored symbols correspond to the individual enzyme molecules. The different lines with the symbols show the tracks of the individual molecules from appearance to the indicated time. Scanning rate, 0.3 s/frame for (a-c), 1 s/frame for (d-g); Scan area, 200 × 200 nm^2 with 100 × 100 pixels for (a-c), 400 × 400 nm^2 with 200 × 200 pixels for (d-g); Z-scale, 10 nm; Bars, 50 nm (a-c), 100 nm (d-g). (h-i) Mean square distance (MSD) plot with line fits (without offset) of ColG (h) and MMP-1 (i) on mica (*open squares*) or collagen microribbon (*open circles*) in TNC (*red*), in TNC containing KCl (*green*: 25 mM (*solid-line squares* in (h) and *solid-line circles* in (i)); 20 mM (*solid-line squares* in (i)); or 30 mM (*dashed-line squares* in (i))) or in TKC (*blue*) buffer. *Dashed-line circles* in (h) correspond to the results from 0.25 s/frame capture speed (0.3 s/frame for others in (h)); *rhombi* in (h) correspond to ColG in the presence of 1 mM *o*-phenanthroline. The estimated diffusion coefficients are shown in **Supplementary Table 3**. The possible maximum noise was estimated from the y-intercept of the line fit to the MSD plot. The square roots of the intercepts were 6.0 nm, 5.5 nm for ColG in the absence/presence of inhibitor and 5.5 nm for MMP-

1. Thus, the movements of collagenase molecules on microribbons are above the possible maximum noise. Note that these values may result not only from residual noise but also from other interactions such as specific interactions between collagen and collagenase molecules.

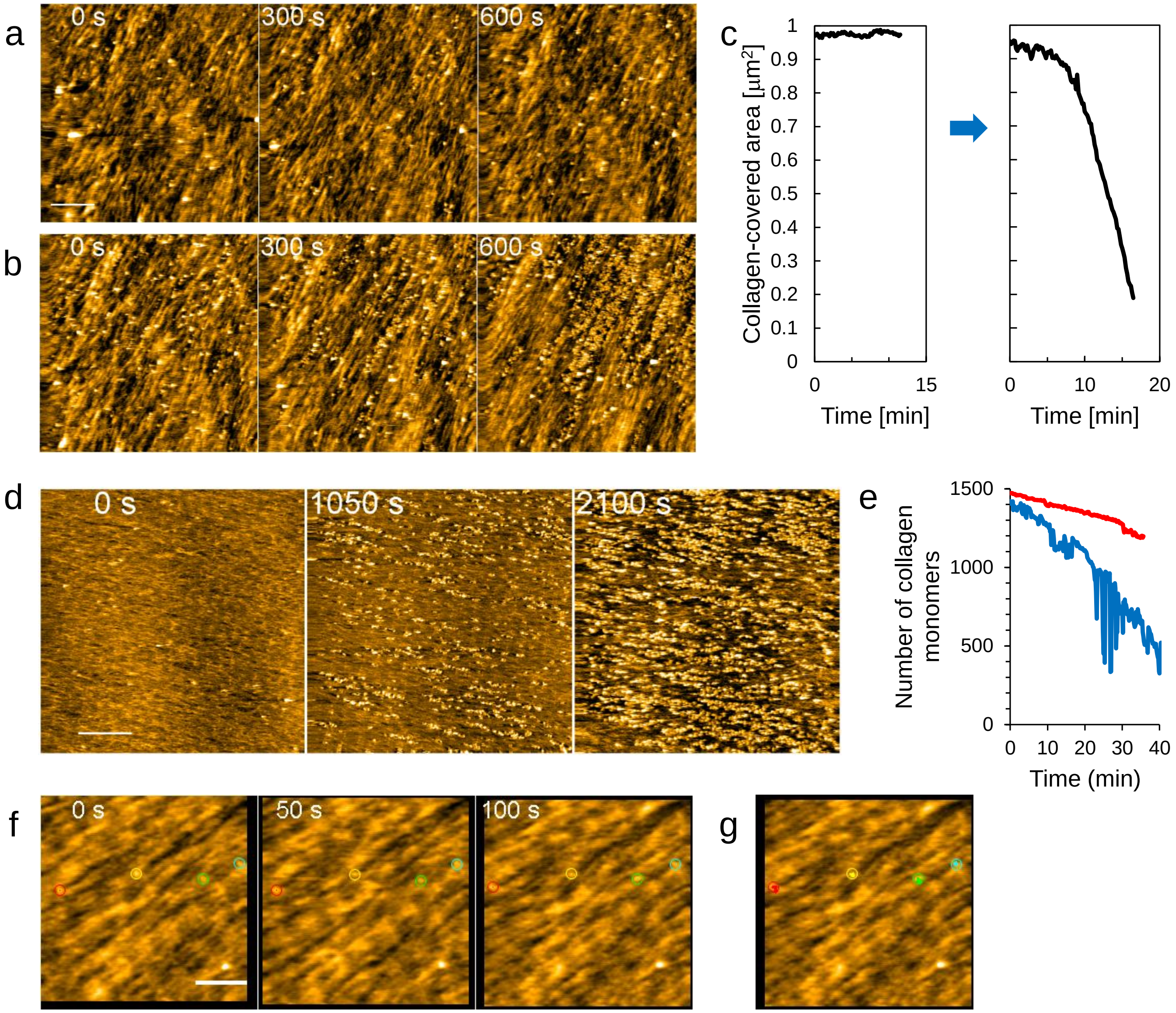

Supplementary Fig. 9. Monovalent cation-dependent collagenase activity and mobility on mica. (a) HS-AFM images after addition of ColG to collagen microribbon in TNC buffer. (b) HS-AFM images of the same area shown in (a) after replacement of the solution with TKC buffer containing ColG. ColG degraded the collagen microribbon (*upper right* in the frame at 600 s). (c) Collagen coverage area over time; ColG did not degrade the collagen fibrils in TNC (*left*) but did in TKC buffer (*right*). (d) HS-AFM images of collagen microribbon degradation by 1.5 μg/mL ColG in TNKC buffer. (e) Number of remaining collagen molecules over time in the presence of 1.5 μg/mL ColG. Different colors correspond to individual experiments. (f) HS-AFM images after addition of MMP-1 to collagen microribbon in TKC buffer. (g) Traces of individual particles indicated by different colors. Scanning rate, 10 s/frame for (a-b, d), 1 s/frame for (f); Scan area, 1000 × 1000 nm^2 with 500 × 500 pixels for (a-b, d), 400 × 400 nm^2 with 200 × 200 pixels for (f); Bars, 200 nm (a and d) and 100 nm (f); Z-scale, 5 nm.

Supplementary Table 1. *Kinetic parameters for ColG from fluorescence-labeled collagen degradation assay*. The parameters were determined by fitting the kinetic measurements shown in Supplementary Fig. 7b with equation (1).

Strep tag (+/-)/buffer	A_1 (a. u.)	k_1 (/s)	A_1 (a. u.)	k_2 (/s)
(-)/TNC	2680 ± 130	0.00214 ± 0.00032	1260 ± 140	0.0154 ± 0.0008
(+)/TNC	2760 ± 70	0.00179 ± 0.00009	1290 ± 40	0.0132 ± 0.0008
(-)/TKC	2630 ± 70	0.00227 ± 0.00036	1240 ± 116	0.0159 ± 0.0004
(+)/TKC	2770 ± 80	0.00176 ± 0.00014	1330 ± 70	0.0129 ± 0.0010
(-)/TNKC	2620 ± 110	0.00219 ± 0.00023	1200 ± 110	0.0154 ± 0.0011
(+)/TNKC	2730 ± 150	0.00192 ± 0.00021	1190 ± 60	0.0134 ± 0.0012

Supplementary Table 2. *Kinetic parameters for prepared MMP-1 activity toward soluble type I collagen*. These values were determined by fitting the data shown in Supplementary Fig. 7f with the Michaelis-Menten model.

Buffers and temperature	K_m (μM)	k_{cat} (/h)
TNC1, 25ºC	2.0 ± 2.2	3.3 ± 3.0
TNC1, 37ºC	1.0 ± 0.7	11 ± 5
TNKC, 25ºC	0.95 ± 0.37	1.9 ± 0.5
TNKC, 37ºC	0.58 ± 0.33	7.8 ± 2.5

Supplementary Table 3. *Diffusion coefficients (nm^2/s) for ColG, MMP-1 and particles in collagen sample in different buffers on mica or collagen*. Diffusion coefficients were estimated from the mean square distance plots shown in Supplementary Figs. 2 and 8.

	On mica			On collagen	
	TNC	TNKC	TKC	TNKC	TKC
ColG	14.9 ± 2.3	201 ± 17	1100 ± 40		88.1 ± 7.3 (0.25 s/frame)<br>66.0 ± 2.3 (0.30 s/frame)<br>37.1 ± 6.2 (10 mM o-phenanthroline)
MMP-1	59.0 ± 0.6	93.0 (20 mM KCl)<br>107 (30 mM KCl)		70.4 ± 6.3	
Particles in collagen sample		1.73 ± 0.28	8.08 ± 0.69		

5. Supplementary Movies.

Supplementary Movie 1. HS-AFM images of growing collagen microribbon (9 μg/mL rat tail collagen). Scanning rate, 10 s/frame; Scan area, 1000 × 1000 nm^2 with 500 × 500 pixels; Bar, 200 nm; Z-scale, 5 nm.

Supplementary Movie 2. HS-AFM images of collagen fibril degradation by 6 μg/mL ColG in the absence of *o*-phenanthroline. Scanning rate, 10 s/frame; Scan area, 1000 × 1000 nm^2 with 500 × 500 pixels; Bar, 200 nm; Z-scale, 5 nm.

Supplementary Movie 3. HS-AFM images of ColG moving on collagen fibrils in the absence of *o*-phenanthroline, shown with (*right*) or without (*left*) collagen polarity (*white line with N and C*), traces (*lines*) and positions (*open circles*) of individual ColG molecules from binding to dissociation. Scanning rate, 0.3 s/frame; Scan area, 200 × 200 nm^2 with 100 × 100 pixels; Z-scale; 5 nm; Bar, 50 nm.

Supplementary Movie 4. HS-AFM images of ColG moving on collagen fibrils in the presence of 1 mM *o*-phenanthroline, shown with (*right*) or without (*left*) collagen polarity (*white line with N and C*), traces (*lines*) and positions (*open circles*) of individual ColG molecules from binding to dissociation. Scanning rate, 0.3 s/frame; Scan area, 200 × 200 nm^2 with 100 × 100 pixels; Z-scale, 5 nm; Bar, 50 nm.

Supplementary Movie 5. HS-AFM images of minimum collagen fibril degradation and rearrangement in the presence of ColG, shown with (*right*) or without (*left*) collagen polarity (*white line with N and C*), traces (*lines*) and positions (*open circles*) of individual ColG molecules from binding to dissociation. Scanning rate, 0.3 s/frame; Scan area, 200 × 200 nm^2 with 100 × 100 pixels; Z-scale; 5 nm; Bar, 50 nm.

Supplementary Movie 6. HS-AFM images of removal of a single collagen fibril accompanied by a single ColG movement, shown with (*right*) or without (*left*) collagen polarity (*white line with N and C*), and positions of the collagen fibril (*arrowheads*) and the ColG molecule (*open circles*). These images are cropped from the HS-AFM image sequence taken at 0.3 s per frame with 200 × 200 nm^2 (100 × 100 pixels). Z-scale, 5 nm. Bar, 20 nm.

Supplementary Movie 7. HS-AFM images of MMP-1 moving on collagen fibrils, shown with (*right*) or without (*left*) collagen polarity (*white line with N and C*), traces (*lines*) and positions (*open circles*) of individual MMP-1 molecules from appearance to disappearance. Scanning rate, 0.3 s/frame; Scan area, 200 × 200 nm^2 with 100 × 100 pixels; Z-scale, 5 nm; Bar, 50 nm.

6. Supplementary References

1. Cisneros, D. A., Hung, C., Franz, C. M. & Muller, D. J. Observing growth steps of collagen self-assembly by time-lapse high-resolution atomic force microscopy. *J. Struct. Biol.* **154,** 232–245 (2006).
2. Jiang, F., Hörber, H., Howard, J. & Müller, D. J. Assembly of collagen into microribbons: effects of pH and electrolytes. *J. Struct. Biol.* **148,** 268–78 (2004).
3. Shoulders, M. D. & Raines, R. T. Collagen structure and stability. *Annu. Rev. Biochem.* **78,** 929–58 (2009).
4. Sarkar, S. K., Marmer, B., Goldberg, G. & Neuman, K. C. Single-molecule tracking of collagenase on native type i collagen fibrils reveals degradation mechanism. *Curr. Biol.* **22,** 1047–1056 (2012).
5. Welgus, H. G., Jeffrey, J. J. & Eisen, A. Z. Human Skin Fibroblast collagenase: Assessment of activation energy and deuterium isotope effect with collagenous substrates. *J. Biol. Chem.* **256,** 9516–9521 (1981).
6. Goldberg, G. I., Strongin, A., Collier, I. E., Genrich, L. T. & Marmer, B. L. Interaction of 92-kDa type IV collagenase with the tissue inhibitor of metalloproteinases prevents dimerization, complex formation with interstitial collagenase, and activation of the proenzyme with stromelysin. *J. Biol. Chem.* **267,** 4583–91 (1992).
7. Aimes, R. T. & Quigley, J. P. Matrix metalloproteinase-2 is an interstitial collagenase. Inhibitor-free enzyme

catalyzes the cleavage of collagen fibrils and soluble native type I collagen generating the specific 3/4- and 1/4-length fragments. *J. Biol. Chem.* **270,** 5872–6 (1995).

8. Welgus, H. G., Jeffrey, J. J. & Eisen, A. Z. The collagen substrate specificity of human skin fibroblast collagenase. *J. Biol. Chem.* **256,** 9511–5 (1981).
9. Welgus, H. G., Jeffrey, J. J., Stricklin, G. P., Roswit, W. T. & Eisen, A. Z. Characteristics of the action of human skin fibroblast collagenase on fibrillar collagen. *J. Biol. Chem.* **255,** 6806–13 (1980).
10. Saffarian, S., Collier, I. E., Marmer, B. L., Elson, E. L. & Goldberg, G. Interstitial collagenase is a Brownian ratchet driven by proteolysis of collagen. *Science* **306,** 108–11 (2004).
